# Social bond dynamics and the evolution of helping

**DOI:** 10.1073/pnas.2317736121

**Published:** 2024-03-07

**Authors:** Olof Leimar, Redouan Bshary

**Affiliations:** ^a^Department of Zoology, Stockholm University, Stockholm 106 91, Sweden; ^b^Institute of Biology, University of Neuchâtel, Neuchâtel 2000, Switzerland

**Keywords:** reciprocity, learning, interdependence, game theory

## Abstract

Empiricists often struggle to apply game theory models to real-life cases of animal cooperation. One reason is that many examples of cooperation occur in stable groups, where individuals form social bonds that influence exchanges of help in ways that are not well described by previous models, including the extent of reciprocity and how relationships are initiated. We present a game theory model exploring the conditions under which social bonds between group members promote cooperation. In the model, bonds build up from exchanges of help in a similar way as the strength of association increases in learning, as in the Rescorla–Wagner rule. The bonds in turn affect partner choice and influence helping amounts. The model has a mechanism of reciprocity for bonded pairs, which can evolve toward either loose or strict reciprocation. Several aspects of the model are inspired by observations of food sharing in vampire bats. We find that small social neighborhoods are required for the evolutionary stability of helping, either as small group sizes, or if bonded members of larger groups can form temporary (daily) smaller groupings. The costs of helping need to be fairly low, while the benefits can be substantial. The form of reciprocity that evolves is neither immediate nor very strict. Individuals in need request help based on bond strength, but there is also an evolved preference for initiating bonds with new group members. In contrast, if different groups come into temporary contact, the evolved tendency is to avoid forming bonds between groups.

## Significance

The search for evolutionary explanations of cooperation between members of social groups has long been a high profile endeavor. A case of particular interest is when individuals develop a network of friends and exchange help through these social bonds. The question of helping between friends was given emphasis at the start of the evolutionary study of cooperation, more than 50 y ago, but it has remained without decisive resolution since that time. Here, we present a game-theory analysis of helping through the build-up of social bonds. We find that there is a form of reciprocity in socially bonded pairs, which is neither immediate nor very strict, and that relatively small social neighborhoods are required for the evolutionary stability of helping.

Over a period of several decades, game-theory modeling has made important contributions to the study of the evolution of cooperation (1). The modeling has shown that strategies of investment into a partner can be evolutionarily stable and result in lifetime direct fitness benefits, verifying in principle that cooperation could evolve. Still, a remaining and even more challenging task is to apply game-theory models to real-life cases of cooperation. A number of empirical examples involve species that live in fairly stable groups, where individuals seldom change group after becoming adult. The individuals typically do not interact randomly with other group members, but tend instead to have one or a few preferred partners. These enduring affiliative relationships are described as social bonds (2, 3). Pairs classified as bonded spend more time in proximity to each other (4), have more favorable interactions, such as body heat sharing (5), grooming (6), coalition formation and food sharing (7), and are more tolerant toward each other (8). The evolution of helping through social bond formation is thus a biologically relevant example of cooperation, but models that explain when this form of helping can evolve are currently lacking. Here, we provide such a model.

There are several challenges facing the modeling, including accounting for individual variation and specifying the cognitive and behavioral mechanisms of bond formation. The bonds should influence decisions about who to request help from and how much help to provide to different partners, while allowing for the possibility of providing no or very little help. In our model, social bonds build up during exchanges of help in a similar way as the strength of association in Pavlovian learning, and individuals have genetically determined bond build-up or learning rates, which can evolve. If bonds build up, individuals in need can use bond strengths to decide who to ask help from, using a choice mechanism with bond strengths as values. Bond strengths can also be used when individuals in a group form temporary (daily) subgroups, which allows for closer association between bonded individuals, provided that this tendency to associate evolves. The tendency (positive or negative) to initiate bonds with new group members, the maximum amount of help provided, and the degree of enforcement of reciprocity within a bond are additional traits in the model. We examine the evolution of these genetically determined traits, using individual-based simulation of large populations of haploid, sexually reproducing individuals.

In our model analyses, we first study how helping with social bonds might operate, in terms of the build-up of bond strength, the helping amounts, the extent of reciprocity, and the degree of association between partners. We then investigate whether there are requirements on the sizes of social neighborhoods (groups and sub-groupings) for this type of helping to be evolutionarily stable. We also investigate how readily new social bonds are initiated, and whether this depends on the type of contacts between individuals, either as new permanent group members, or as temporary contacts between members of different groups. To avoid any effects of relatedness on helping, new recruits into a group are derived from parents chosen from the entire population.

Selection operates through differential survival, with helping in the form of food sharing that leads to survival costs and benefits for donor and recipient individuals. In this way, individuals in bonded pairs, as well as the members of small groups, can become dependent on each other for survival. The model is inspired by observations of food sharing in vampire bats (9), for which there are data on the extent of reciprocity (10, 11).

After presenting the model and results, we discuss the similarities and differences between our analysis here and previous game-theory-inspired ideas about helping in groups (12–17), including the concept of raising-the-stakes cooperation (18, 19) and the formation of social relationships (20). We emphasize the value of implementing sufficient details of the cognitive and behavioral mechanisms operating in real-life examples when attempting to explain the evolution of cooperation.

## Model and Results

Here, we give an overview of the model, with a detailed description in the *Materials and Methods* and *SI Appendix*. There is large population (of size at least 4,000), and recruited offspring have parents randomly selected from the population, but adult individuals spend their lives in smaller groups of size *N*. Time is divided into steps, where a time step is 1 d. A group can be structured into temporary subgroups, which we identify with places, for instance resting places. For vampire bats, a resting place might be a daytime roost (9), but in practice, group members could form subgroups in some other way. There are *K* subgroups for a group, with on average G=N/K individuals per subgroup, and each day each group member can select which subgroup to join. If two individuals are in the same subgroup, they can help each other.

We use a state variable $z_{i}$ to indicate the current (nightly) foraging success of individual *i*. The state is $z_{i}=1$, corresponding to success in foraging, with probability $p_{\text{s}i}$ and $z_{i}=0$ (failure in foraging) with probability $1-p_{\text{s}i}$. An individual’s probability of foraging success depends on its phenotypic quality $q_{i}$. Only individuals with $z_{i}=0$ ask for help on a given day. They only ask help from other individuals *j* they perceive have a state of $z_{j}=1$, and they have a probability $p_{\text{d}}$ of correctly detecting that state.

### Bond Strength.

We use the symbol *x* to denote the strength of a bond. For individuals *i* and *j* that donate or receive help on a given day, there is an updating of bond strengths. The starting value of the bond strength is $x_{\text{s}}$, which should be large enough that at least some help is donated early in a partnership (we used $x_{\text{s}}=1.25$; in a real case, some behavior like grooming might give the starting value). After a request from *i* to an individual *j*, *i* updates its value $x_{\mathrm{ij}}$ using a genetically determined learning rate $\alpha_{i}$ and an asymptotic (maximum) bond strength $x_{\text{a}}$. If $u_{\mathrm{ji}}$ is the amount of help from *j* to *i*, the update is

[1] $$\begin{array}{c} x_{\mathrm{ij}}^{\prime }=x_{\mathrm{ij}}+\alpha_{i}u_{\mathrm{ji}}\frac{x_{\text{a}}-x_{\mathrm{ij}}}{x_{\text{a}}-x_{\text{s}}}, \end{array}$$

where $x_{\mathrm{ij}}^{\prime }$ is the updated value of $x_{\mathrm{ij}}$. The donating individual *j* also updates its bond strength $x_{\mathrm{ji}}$, as follows:

[2] $$\begin{array}{c} x_{\mathrm{ji}}^{\prime }=x_{\mathrm{ji}}+\alpha_{j}u_{\mathrm{ji}}\frac{x_{\text{a}}-x_{\mathrm{ji}}}{x_{\text{a}}-x_{\text{s}}}\text{.} \end{array}$$

**Fig. 1.**
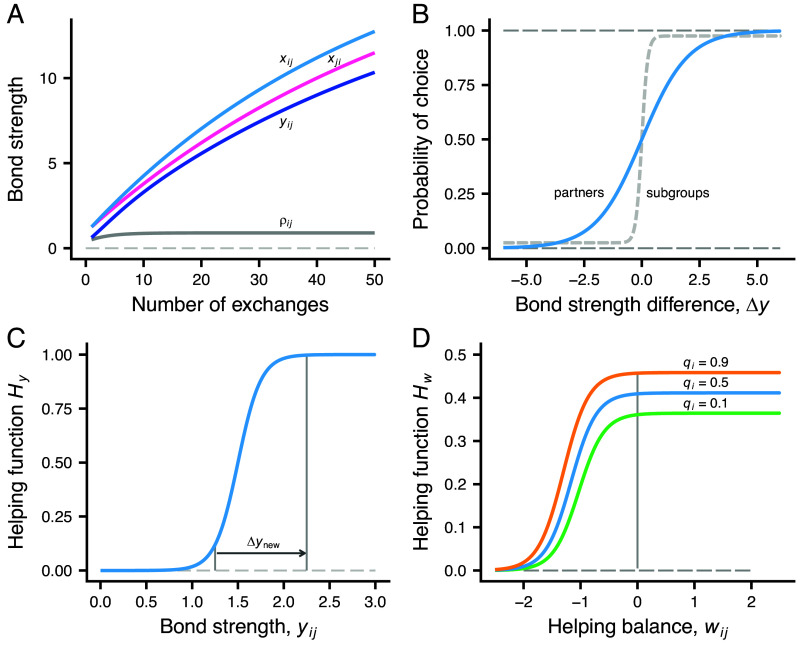
Model elements. (*A*) Stylized bond strength dynamics. In the model, the bond strength $x_{\mathrm{ij}}$ between individuals *i* and *j*, as perceived by *i*, increases with each helping exchange. Individuals have genetically determined learning rates $\alpha_{i}$ and $\alpha_{j}$ which, together with the helping amounts *u*, determine the bond strength increments. The light blue and red curves show $x_{\mathrm{ij}}$ and $x_{\mathrm{ji}}$, assuming $\alpha_{i}=1.2$ and $\alpha_{j}=1.0$ and all helping amounts u=0.3. The dark blue curve shows the effective bond strength $y_{\mathrm{ij}}$, which is $x_{\mathrm{ij}}$ multiplied by an estimate $\rho_{\mathrm{ij}}$ of association between *i* and *j* ($0\leq \rho_{\mathrm{ij}}\leq 1$), which indicates how often *i* and *j* have been in the same subgroup in the recent past (the gray curve is an illustration of how $\rho_{\mathrm{ij}}$ might increase over time). (*B*) Soft-max probabilities of choice by an individual between two alternatives, as a function of the difference Δy in effective bond strength. The blue/dashed light gray curves give the choice between two partners/subgroups. (*C*) The dependence of the helping function $H_{y}$ on the effective bond strength $y_{\mathrm{ij}}=\rho_{\mathrm{ij}}x_{\mathrm{ij}}$. The arrow illustrates a possible extra effect of interacting with a new partner. (*D*) The dependence of the helping function $H_{w}$ on the helping balance $w_{\mathrm{ij}}$ between individuals *i* and *j* (the curves are for $z_{i}=1$, different values of $q_{i}$, and $h_{\text{a}i}=0.47$, $h_{\text{s}i}=4.5$). The amount *u* of help donated is the product $u=H_{w}H_{y}$ of the two functions.

By having both *i* and *j* update their bond strength values, there is approximate symmetry in the bond dynamics. An idea behind this is that the evolutionary origin of social bonds might be mother-offspring relations, where helping is, at least initially, in only one direction, but the bond is bi-directional. The form of the bond strength dynamics in Eqs. **1** and **2** is inspired by the Rescorla–Wagner model of classical conditioning (21) and is illustrated in Fig. 1*A*.

To account for the recent subgroup association between individuals, we introduce an effective (association adjusted) bond strength $y_{\mathrm{ij}}$, defined as the product of an estimated association $\rho_{\mathrm{ij}}$ and the bond strength $x_{\mathrm{ij}}$. The estimate $\rho_{\mathrm{ij}}$ increases or decreases at a certain rate per period, depending on whether or not *i* and *j* are in the same subgroup, and thus estimates the subgroup association, whereas $x_{\mathrm{ij}}$ represents the accumulated exchange of help between *i* and *j*. So, for *i*, the effective strength of the bond to *j* is $y_{\mathrm{ij}}=\rho_{\mathrm{ij}}x_{\mathrm{ij}}$. This effective bond strength can decrease over time, which happens if previously bonded individuals are mostly in different subgroups.

For an individual in need, if there is more than one other individual to ask help from, the effective bond strengths are used as values for a soft-max choice, which is illustrated in Fig. 1*B* for a choice between two alternatives. Evolutionary changes in the learning rates $\alpha_{i}$ will influence such differences in bond strength, so this partner choice mechanism can be modified by evolution. Furthermore, the daily choices of subgroup to join are influenced by an individual’s recent experience of the average effective bond strengths to other individuals encountered in different subgroups. To study the evolution of the choice of subgroup, we assume that individuals have a genetically determined trait $\beta_{i}$ that influences their sensitivity to subgroup differences in estimated average bond strength ($\beta_{i}$ can be positive, zero, or negative). An illustration of the resulting probability of choice between two subgroups appears in Fig. 1*B*.

An individual in need can ask for help from more than one of the available group members. One reason for introducing this is that it occurs in vampire bats (10). In the model, individuals in need have the opportunity to ask for help twice, from different individuals among the available ones in the current subgroup.

### Determination of Helping Amounts.

The amount $u_{\mathrm{ij}}$ donated by *i* when *j* asks for help depends of several factors. First, there is a sigmoid dependence on the effective bond strength $y_{\mathrm{ij}}$, which is shown as the helping function $H_{y}$ in Fig. 1*C*. This form of dependence allows in principle for successive increases in the helping amounts, as in raise-the-stakes cooperation (18). To further investigate how natural selection operates on how much help is given to a new recipient, we introduce a genetically determined trait $\Delta y_{\text{new}i}$, which acts as a temporary increment to the effective bond strength on the first occasion of donating to an individual (illustrated by the arrow in Fig. 1*C*). The increment can evolve to be positive, zero, or negative, and is also applied to new individuals when choosing who to ask help from (Fig. 1*B*).

The model has another helping function $H_{w}$, illustrated in Fig. 1*D*. The amount of help donated by *i* to *j* is the product of the two functions, $u_{\mathrm{ij}}=H_{y}H_{w}$. The function $H_{w}$ determines the maximum amount of help and the extent of reciprocity, through two genetically determined traits $h_{\text{a}i}$ and $h_{\text{s}i}$, with $h_{\text{a}i}$ being the maximum amount of help possible from a highest quality individual ($q_{i}=1$) and $h_{\text{s}i}$ determining the sensitivity to the total helping balance $w_{\mathrm{ij}}$. The total helping balance is the total previously received minus donated for *i* interacting with *j*. Provided that $h_{\text{s}i}$ evolves to a positive value, it follows that if $w_{\mathrm{ij}}$ becomes too negative, the amount *i* donates to *j* becomes small and approaches zero (Fig. 1*D*). Further details about the helping functions are given in *Methods*.

### Mortality.

**Fig. 2.**
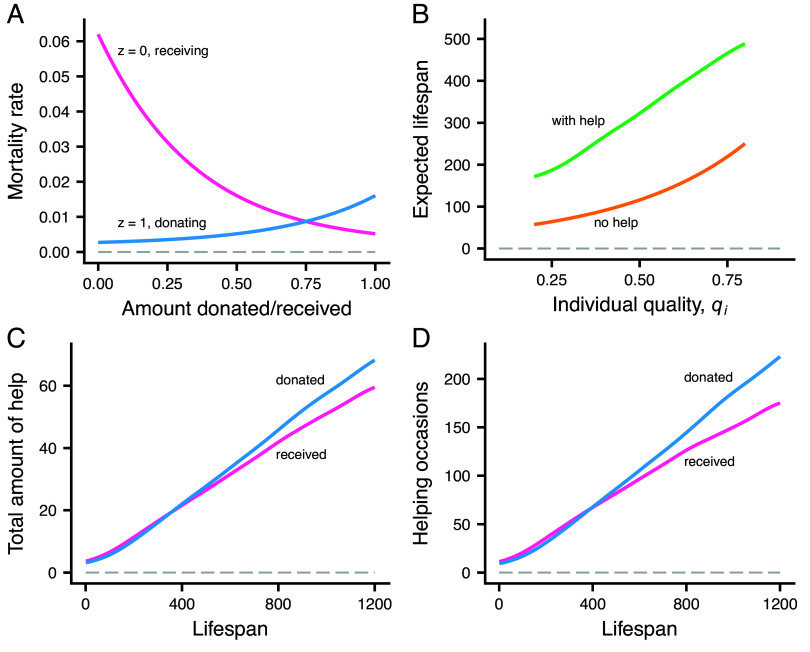
Mortality and lifespan in the model. (*A*) Day-to-day mortality for successful foragers (z=1) that donate help (blue) and unsuccessful foragers (z=0) that receive help (red), as a function of the amount transferred (i.e., the change in resource balance *ζ*). (*B*) Effects of individual phenotypic quality $q_{i}$ on expected life span, with and without helping (see *SI Appendix*, Fig. S1*A* for the distribution of $q_{i}$). (*C*) Average total amounts of help donated (blue) and received (red) by an individual, as a function of the individual’s lifespan. (*D*) Average total number of times help was donated (blue) and received (red) by an individual. The curves are kernel smoothing fits and are based on ca 12,000 individuals with complete life histories. There is considerable variation around the fitted curves (*SI Appendix*, Fig. S1). The data for panels (*B*) to (*D*) come from a simulation with 175 groups of size N=24, where each group has access to K=6 subgroups (places, G=4). The simulation was run at evolutionary equilibrium for 4,000 d, and the data come from individuals born in the earlier part of this interval, thus including those with very long lifespans.

The day-to-day mortality depends on an individual’s foraging success ($z_{i}$), but also on its resource balance $\zeta_{i}$ for a given day (Fig. 2*A*). The current day resource balance increases when an individual receives help and decreases when it donates. At the start of a day, $\zeta_{i}$ is assumed to depend on the phenotypic quality $q_{i}$, such that a higher quality individual suffers a smaller mortality increase when donating a certain amount. A consequence of the dependence illustrated in Fig. 2*A* is that the survival cost of donating starts out small but accelerates for larger amounts, whereas for receiving there is first a sharp decrease in mortality, which flattens out for larger amounts.

Individuals who die are replaced through reproduction by surviving individuals. Replacement happens at intervals of *T* periods (we used T=20). To avoid effects of local relatedness, parents to new individuals are randomly drawn from the global population.

### Examples of Helping with Social Bonds.

To illustrate helping in the model, we present evolutionary equilibrium results for a case with groups of size N=24 and K=6 subgroups (G=4). Fig. 2*B* shows that helping substantially increases the expected lifespan compared to a hypothetical case where there is no helping, with a proportionally greater increase for lower-quality individuals. The average number of times and total amounts for donating and receiving help increase approximately in proportion to life span (Fig. 2 *C* and *D*). Higher quality individuals tend to live longer and on average have relationships where the partner needs help more than they themselves do, which explains the divergence between donated and received help for long lifespans. In the simulation, an average-quality individual needs help on one out of every 10 d.

**Fig. 3.**
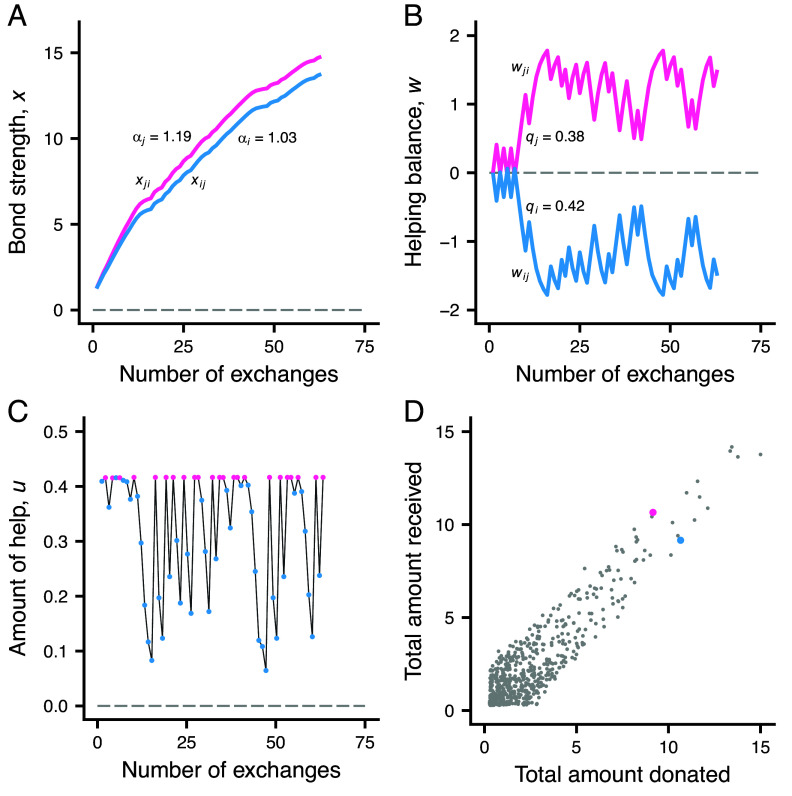
Example of bond dynamics and helping for a pair *i* and *j* with many helping exchanges. In the panels, blue and red indicate *i* and *j*. (*A*) The build-up of bond strength $x_{\mathrm{ij}}$ for *i* and $x_{\mathrm{ji}}$ for *j*. The genetically determined bond strength learning rates $\alpha_{i}$ and $\alpha_{j}$ appear near the corresponding curves. (*B*) The total helping balance $w_{\mathrm{ij}}$ for *i* and $w_{\mathrm{ji}}$ for *j*, together with the phenotypic quality values $q_{i}$ and $q_{j}$ for the example. (*C*) The amounts of help $u_{\mathrm{ij}}$ and $u_{\mathrm{ji}}$ donated by one individual to the other (the trait for helping amounts are $h_{\text{a}i}=0.49$ and $h_{\text{a}j}=0.49$). (*D*) The color-coded points show the total amounts donated and received between *i* and *j*. The gray points show a sample of 1,000 partnerships from those with helping at least once in each direction. The data come from the same simulation as in Fig. 2.

An example of the exchange of help between two individuals appears in Fig. 3, coming from the same simulation as in Fig. 2. The build-up of bond strength in Fig. 3*A* is less smooth than for the stylized case in Fig. 1*A*. The reason is that the helping balance *w* varies (Fig. 3*B*), in this example in such a way that one of the individuals mostly has a negative balance (i.e., donating more than receiving; blue curve), causing the individual to reduce the amount it donates when the other asks for help (Fig. 3*C*). The individual with mostly negative helping balance still donates a larger total amount than it receives (blue point in Fig. 3*D*), because of the greater number of times the other asks for help. In general, a lower-quality individual will ask for help more often, and there is also substantial random variation in how often individuals fail in foraging and need help. A random sample of relationships from the simulation illustrates that there is notable reciprocity in the relationships (gray points in Fig. 3*D*; the Spearman correlation between total donated and received is $r_{\text{S}}=0.77$ for this simulation), but it is still common that a lack of strict reciprocation gives rise to a negative helping balance for one of the partners, causing a reduction in the amount it donates (cf. Fig. 1*D*), and this is the reason for the band-like pattern in Fig. 3*D*.

A striking aspect of the pattern of helping seen in Fig. 3*C* is that individuals donate close to their maximum amounts at the start of a relationship. The reason is that the bond strength increment (trait $\Delta y_{\text{new}}$, Fig. 1*C*) evolved to a large positive value (see *SI Appendix*, Table S1 for evolutionary equilibrium trait values). As a consequence, individuals have a strong tendency to initiate relationships with new permanent group members, both in terms of requesting help and amounts donated, and do not follow a raise-the-stakes procedure (but see below for “visitor” individuals).

**Fig. 4.**
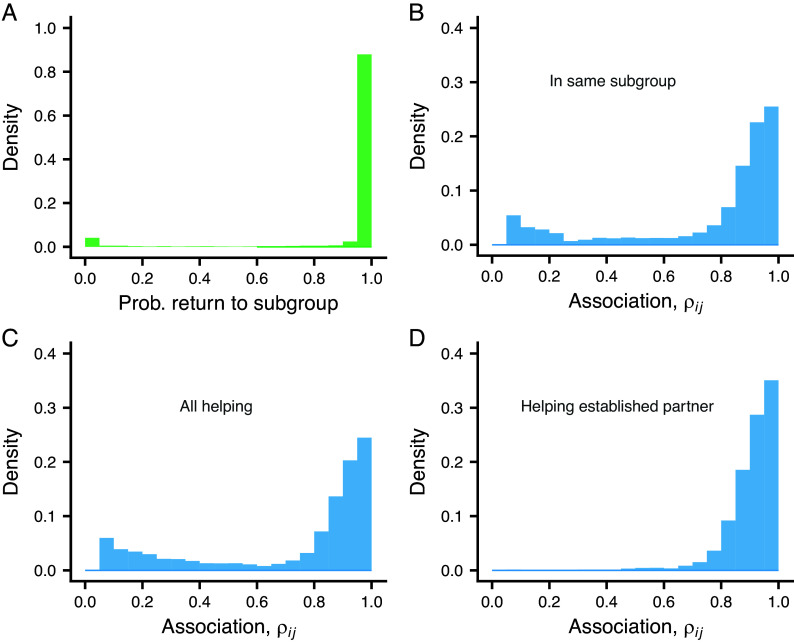
The degree of subgroup preference shown by individuals, measured as their probability to return to the current subgroup (place) in the next period, and the distribution of estimates $\rho_{\mathrm{ij}}$ of associations between individuals, for different samples of pairs *i* and *j*. (*A*) Distribution of the probability to return to the current subgroup in the next period, shown as a density histogram. (*B*) Distribution of $\rho_{\mathrm{ij}}$ for individuals in the same subgroup, regardless of helping interactions. (*C*) Distribution for all helping interactions. (*D*) Distribution for helping between established partners, in the sense of recipient and donor having previously exchanged help in both directions. The data come from the same simulation as in Fig. 2.

In our model, a group has a subgroup structure of different places. A strong tendency to prefer subgroups with higher average bond strength evolved in the simulation (*SI Appendix*, Table S1). As a consequence, the probability that an individual is found the next day in its current subgroup (i.e., its current place) is concentrated near 1.0 (Fig. 4*A*). The small bump near zero in Fig. 4*A* is explained by the model assumption that an individual ends up in a random place with a small probability (0.05). Such individuals will mostly not return there the next day (this is meant to represent variability in the particular ways that individuals distribute themselves over subgroups on a given day, and allows individuals to encounter all group members).

An individual *i* has an estimate $\rho_{\mathrm{ij}}$ of its subgroup-association to another individual *j*. Fig. 4*B* shows the distribution of these estimates between randomly chosen pairs from the same subgroup. Acts of helping may occur between pairs of individuals in the same subgroup, including both new and established partners, making the distribution of $\rho_{\mathrm{ij}}$ for pairs where there is help (Fig. 4*C*) similar to that for randomly chosen pairs in the subgroup. In contrast, for helping between established partners (i.e., in pairs with previous helping in both directions), the distribution contains mostly high association values (Fig. 4*D*).

### The Size of the Social Neighborhood.

**Fig. 5.**
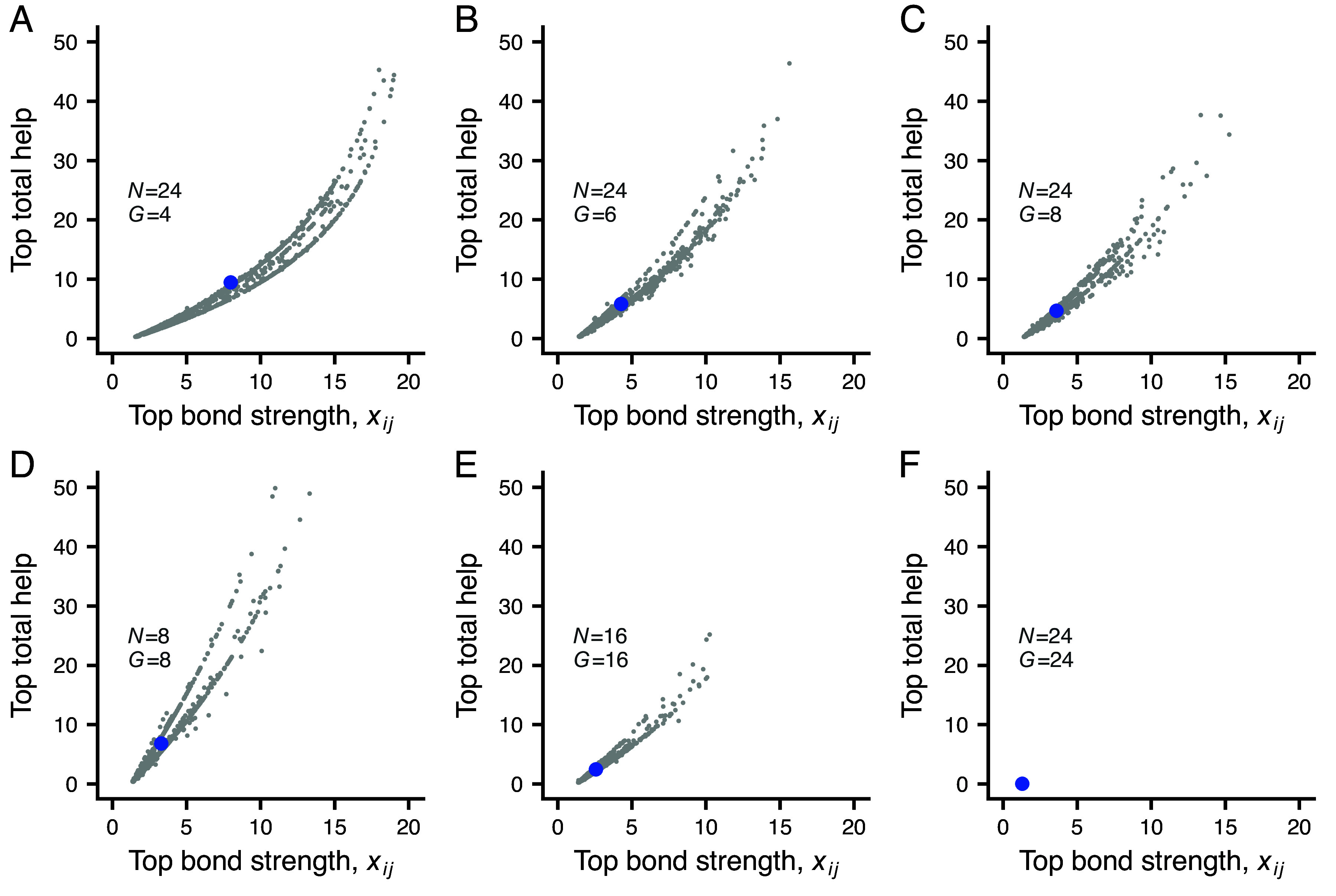
Effects of the size of the social neighborhood on bond formation and helping. The top bond strength for an individual is the highest value of $x_{\mathrm{ij}}$ among the partnerships the individual has initiated over its lifetime, and the top total help is the sum of all help donated and received in this top partnership. The gray points in the panels show random samples (of size 1,000) of individuals from evolutionary simulations, and the larger blue points show the sample means. Each simulation has a population size of either 4,200 or 4,000, split into groups of size *N*. Each group has one or more subgroups in which individuals can associate, and *G* is the expected number of individuals in a subgroup. Panels (*A*–*C*) show cases with group size N=24, split up into 6, 4, or 3 subgroups (so that G=4,6,8), and panels (*D*–*F*) show cases with different group sizes N=8,16,24 and a single subgroup (so that G=N). Note that helping evolved to near zero in panel (*F*). Each simulation was run at evolutionary equilibrium for 4,000 periods, and the random samples are of individuals born in the earlier part of this interval.

A relatively small size of the social neighborhood is needed for substantial helping to evolve in our model (Fig. 5). For larger group sizes *N*, the social neighborhood can be a subgroup with on average a small number *G* of individuals (Fig. 5*A*–*C*), or it can be a smaller group with a single subgroup (Fig. 5*D*). For larger social neighborhoods, the trait $h_{\text{a}i}$, giving the maximum amount donated, evolves to a small value, resulting in little helping, as illustrated in Fig. 5*F*. See also *SI Appendix*, Figs. S2 and S3 for illustrations of the total amounts received and donated for the cases in Fig. 5, and *SI Appendix*, Fig. S4 for the corresponding distributions of an individual’s lifetime number of partners.

Part of the explanation for the reduction or elimination of helping with larger social neighborhoods is that individuals evolve a strong tendency to initiate bonds with new group members (the trait $\Delta y_{\text{new}}$ evolves to a large positive value; *SI Appendix*, Table S1). Individuals thereby gain the short-term advantage of a large network of potential partners, but this has the evolutionary consequence of reducing the amount of help donated to a partner. The explanation can be checked by fixing the trait $\Delta y_{\text{new}}$ at a negative value, causing individuals to prefer to request help from and donate help to established partners, and then see whether helping evolves. The result is that notable helping with social bonds evolves for the group size in Fig. 5*F*, which is illustrated in *SI Appendix*, Figs. S5 and S6 (see also *SI Appendix*, Table S1). In this situation, the bond strength learning rates are high and relationships show an initial increase in helping amounts, corresponding to raising the stakes (*SI Appendix*, Fig. S6).

To further investigate evolution of the tendency to initiate new bonds, we developed a variant of the model where individuals from one group sometimes visit another group, but return to their original group after a number of days (see *SI Appendix* for details). Evolutionary simulations for this model variant show that individuals avoid forming bonds between groups, but still evolve a strong tendency to initiate bonds with new within-group individuals (*SI Appendix*, Table S2). As a consequence, essentially no help is exchanged between members of different groups (*SI Appendix*, Fig. S7).

### Marginal Costs and Benefits.

Helping amounts in our model are quantitative variables, so we can compute the increase or decrease in mortality from a slight change in the amount of help donated, for any particular situation (*SI Appendix*). Performing this for the hypothetical case of exceedingly small amounts of help, we find a large marginal benefit–cost ratio of around 80. For the case illustrated in Fig. 3, with amounts around u=0.4, the marginal benefit–cost ratio is around 8 for average-quality individuals. These are high benefit–cost ratios and are typical for our model of helping with social bonds.

### Survival Interdependence, Reciprocity, and Partner Choice.

One explanation for the evolution of helping is that individuals depend on each other for help when help is needed. As a consequence, they would have an interest in promoting each other’s welfare. The interdependence would be strongest in small groups and become diluted for larger group sizes. Another explanation is that reciprocity promotes helping, so that individuals help in order to receive reciprocal help when they need it. For our model, reciprocity is more important than interdependence in promoting the evolution of helping. For the smallest possible group size of N=2, substantial helping evolves when reciprocity operates, but there is very little helping when reciprocity is prevented, by fixing the trait $h_{\text{s}i}$ at zero (compare cases 9 and 10 in *SI Appendix*, Table S3).

The concept of a social bond presupposes individual recognition, but one can still investigate what happens to the evolution of helping if an individual treats all group members as the same, as in generalized reciprocity (22). Implementing this in the model (*SI Appendix*), and performing evolutionary simulations of situations with group and subgroup sizes as those Fig. 5, the maximum help trait $h_{\text{a}i}$ evolved to near zero, except for the case with a small group size of N=8 (*SI Appendix*, Fig. S8 and Table S3). Thus, individual recognition is important for the evolution of helping with social bonds. Comparing *SI Appendix*, Fig. S3 with Fig. S8, an interpretation is that partner choice with social bonds contributes to the evolution of helping. Thus, partner choice and reciprocity appear to be the two main mechanisms promoting the evolution of helping in our model.

Finally, we investigated the evolutionary effects of the number of times individuals in need can ask for help and the treatment of a new partner, mainly finding moderate changes in the bond-strength learning rate (*SI Appendix*, Table S4).

## Discussion

Behavioral mechanisms are the main ingredients in our model of helping with social bonds, and we study the evolution of traits that influence these mechanisms through individual-based simulation. An important mechanism is the dynamics of social bonds, which we describe as a psychological process qualitatively similar to associative learning, with a learning-rate trait *α*. At present, the psychology of social bonding is not well enough developed to settle if the analogy to learning is exact, or if bond formation is a similar but separate psychological mechanism. The social bond dynamics in our model has an added element that determines how potential new partners are handled. This element (the trait $\Delta y_{\text{new}}$, Fig. 1*C*) influences the growth and eventual size of an individual’s social network. We found that there is selection for individuals to incorporate new (permanent) group members into their social network ($\Delta y_{\text{new}}$ evolved to positive values; *SI Appendix*, Table S1), and to be generous at the start of a relationship (Fig. 3*C*). This possibility was mentioned by Trivers when discussing the setting up of relationships in humans (12) (we also found situations where individuals avoid initiating bonds with short-term visitors; *SI Appendix*, Fig. S7 and Table S2). At the same time, we found that there is selection for individuals to choose to spend time in subgroups (places) where they are likely to encounter their socially bonded partners and thus maintain the association to these partners (the subgroup choosiness trait *β* evolved to positive values; Figs. 1 and 4 and *SI Appendix*, Table S1). Our analysis thus identifies a tension between, on the one hand, gaining security by maintaining and strengthening existing bonds and, on the other hand, gaining security by enlarging the social network, which is in accordance with the so-called social bet-hedging hypothesis (23).

The tension is responsible for a limitation on the evolution of helping with social bonds that we identified. We found that small social neighborhoods are required (Fig. 5), because for large social neighborhoods, the maximum level of helping (the trait $h_{\text{a}}$) evolves to a small value. In situations with small social neighborhoods, where helping through social bond formation can evolve, we found that a combination of reciprocity, by way of the trait $h_{\text{s}}$, and partner choice based on the strength of social bonds maintains helping (this follows by comparing situations with and without individual recognition: *SI Appendix*, Fig. S3 vs. Fig. S8).

Our model is inspired by observations on female vampire bats (9, 24–26), specifically our assumptions that food donations go from successful to unsuccessful foragers (with a fairly high chance of being successful), that individuals in need can request help more than once, that groups are structured into one or more subgroups (sharing day roosts), and that the survival benefits and costs of food donations have approximately exponential shapes (Fig. 2*A*). For simplicity, the model does not account for the sex differences observed in vampire bats, but instead assumes that all individuals are equally involved in food sharing. The natural within-group recruitment of female offspring is also not implemented in the model, which instead assumes offspring recruitment from the entire population, in order to prevent evolutionary effects of relatedness from contributing to our results.

We might then compare the qualitative results from our model with field and experimental observations on female vampire bats. First, for estimates of subgroup associations, there is the similarity that food-sharing pairs tend to have high association; compare Fig. 4 with (9, 27, 28). Second, our analysis revealed a form of reciprocity that is neither immediate nor very strict (Fig. 3), and this also seems to hold for reciprocity in female vampire bats (10). Third, our finding of an evolved tendency for individuals to incorporate new permanent group members into their social network is in line with experiments showing that nonkin food sharing by female bats expands their social network, which reduces their future risk of not receiving help when they need it (23, 29), and this might explain the evolution of nonkin food sharing (30). Finally, we did not model the fusion of different groups, but the results from our model version with short-term visitors between groups shows some similarity to the experimental fusion of two groups of bats (19) (currently it is not known whether fusions occur in the wild). Specifically, we found that group members avoid forming bonds with visitors (*SI Appendix*, Fig. S7 and Table S2), and we also analyzed a special case of a negative $\Delta y_{\text{new}}$ (*SI Appendix*, Fig. S6 and Table S1),

While our current results (Fig. 3*C*) do not support the idea of raising-the-stakes cooperation (18, 19), different assumptions about how new individuals are recruited into groups might change this. Raising-the-stakes could be interpreted as a form of defense against potential exploiters that request help and then move on to exploit other individuals. As there will be costs associated with delaying the exchange of help, there needs to be a noticeable presence of such exploiters for a strategy of initial caution to be evolutionarily stable (see ref. 31 for a discussion of this point).

There is one previous evolutionary model investigating how individuals might form social bonds when deciding on the distribution of help to other group members (20). The work uses the idea of a Bayesian update (of the probability of donating help when requested) as inspiration for a social bond mechanism. There is some similarity to our approach to social bond dynamics, in that both models describe the build-up of bond strength, but there are also major differences, for instance, in how the helping of individuals in need is implemented (e.g., from our analysis, new permanent group members receive substantial help, Fig. 3*C*, which is not the case for the analysis in ref. 20). Another major difference is that the maximum amount of help to a partner is an evolving trait in our model, underpinning our conclusion that small social neighborhoods are required for substantial helping to evolve, whereas the total amount of help per period donated by an individual was fixed, even for large group sizes, in the model in ref. 20.

More generally, social bonds are found in several groups of mammals and some birds (2, 3, 32, 33). Primate social bonds are the most studied, and an overall conclusion is that bonded (socially integrated) individuals have advantages in terms of health, survival, and reproduction (2, 3). The acts of helping tend to be low-cost behaviors (34), while the advantages of receiving help can be substantial, which holds for our results.

The study of the evolution of cooperation is a very large field, in which a range of ideas continue to be evaluated (17). The importance of reciprocity for helping in social groups is one of the most discussed of these ideas, first dealt with by Trivers more than 50 y ago (12). While immediate and strict reciprocity appears to be rare (35), there is still the possibility that reciprocity operates between group members over a longer time frame (16, 34), and this is broadly in agreement with our results. There are several other important possibilities that we did not include in our analysis, for instance, relatedness and indirect benefits (17), the punishment of defectors (36), and the abandonment of less profitable interactions (37). The focus of our model is on the particular issue of how helping with social bonds might operate and evolve.

In contrast to traditional analyses of cooperation, such as those based on the iterated Prisoner’s Dilemma, our approach is to incorporate specific and potentially realistic cognitive and behavioral mechanisms into game-theory models. This approach is a development of game theory in biology (1, 38). It introduces a certain complexity of assumptions about traits, mechanisms, and life histories, but it has the crucial advantage of bringing modeling and observation into closer contact. In our view, this is essential for progress in the challenging study of social interactions, including the evolution of cooperation in social groups. If we are to understand complex phenomena such as social bonds from an evolutionary point of view, we need game theory that examines the mechanistic underpinnings of decision-making.

## Materials and Methods

### Model Description.

An individual *i* has five genetically determined traits: $\alpha_{i}$ (bond strength learning rate), $\beta_{i}$ (subgroup bond strength sensitivity), $\Delta y_{\text{new}i}$ (bond strength increment for new partner), $h_{\text{a}i}$ (asymptotic helping amount), and $h_{\text{s}i}$ (helping balance sensitivity). Evolutionary equilibrium values of these traits for different simulations appear in *SI Appendix*, Tables S1–S4, together with a full and detailed model description, and with notation for the model in *SI Appendix*, Table S5. In order to allow mutations to efficiently explore the multidimensional trait space, individuals are assumed to be haploid, reproducing sexually such that two parents form a diploid that then produces a haploid offspring, with mutation and recombination. Mutational distributions have long-tailed, Laplacian distributions (*SI Appendix*). As is know from theoretical population genetics (39), the equilibrium mutation-drift-selection trait dynamics typically show fluctuating patterns with substantial auto-correlation over time. For our simulations, this is illustrated in *SI Appendix*, Figs. S9 and S10.

For an individual in need (foraging success $z_{i}=0$), if there is more than one other individual available and estimated to have succeeded in foraging ($z_{j}=1$), the individual chooses to ask help from *j* with a probability proportional to $\exp(y_{\mathrm{ij}})$, where $y_{\mathrm{ij}}$ is the effective bond strength to *j*. For a choice between two individuals, with a bond strength difference of Δy between them, the probability of choice then becomes

[3] $$\begin{array}{c} \frac{1}{1+\exp(-\Delta y)}, \end{array}$$

which is illustrated in Fig. 1*B*. Choices between subgroups (places) are similarly influenced by an individual’s estimate of the average effective bond strength to other individuals encountered in different subgroups. There is a small probability $\epsilon_{\text{p}}$ that the individual ends up in a random subgroup (we used $\epsilon_{\text{p}}=0.05$), and otherwise the probability to choose subgroup *k* is proportional to $\exp(\beta_{i}{\hat{y}}_{\mathrm{ik}})$, where ${\hat{y}}_{\mathrm{ik}}$ is the estimate. For two subgroups to choose between, and with Δy the difference between them in estimated average bond strength, the probability of choice becomes

[4] $$\begin{array}{c} \frac{1-\epsilon_{\text{p}}}{1+\exp(-\beta_{i}\Delta y)}+\frac{1}{2}\epsilon_{\text{p}}, \end{array}$$

which is illustrated as a dashed line in Fig. 1*B* (with $\beta_{i}=7.9$; see *SI Appendix* for further details, including a density dependence of the choice).

The amount of help individual *i* donates when *j* request help depends on several factors, and is expressed as a product $u_{\mathrm{ij}}=H_{y}H_{w}$. The dependence on the effective bond strength is

[5] $$\begin{array}{c} H_{y}=\frac{1}{1+\exp(-d(y_{\mathrm{ij}}-y_{0}))}, \end{array}$$

where *d* and $y_{0}$ are parameters (we used d=8.0 and $y_{0}=1.5$). This is illustrated in Fig. 1*C*, including the effect $\Delta y_{\text{new}i}$ of donating for the first time to new individual. There is also a dependence on the helping balance $w_{\mathrm{ij}}$ with *j*, as well as on the quality $q_{i}$, the foraging success state $z_{i}$, and the current resource balance $\zeta_{i}$ of *i*, given by

[6] $$\begin{array}{c} H_{w}\left(q_{i},w_{\mathrm{ij}},z_{i},\zeta_{i}\right)=\frac{h_{\text{a}i}(g_{0}+\left(1-g_{0}\right)q_{i})}{1+\exp(-h_{\text{s}i}w_{\mathrm{ij}}-bz_{i}-c\zeta_{i})}, \end{array}$$

where $h_{\text{a}i}$ and $h_{\text{s}i}$ are genetically determined traits and $g_{0}$, *b* and *c* are parameters ($g_{0}=0.75$; *b* and *c* also appear in the mortality function below). The function $H_{w}$ is illustrated in Fig. 1*D*. Finally, the day-to-day mortality rate for an individual *i* is

[7] $$\begin{array}{c} \mu \left(z_{i},\zeta_{i}\right)=\frac{1-\mu_{0}}{1+\exp(a+bz_{i}+c\zeta_{i})}+\mu_{0}, \end{array}$$

where *a*, *b*, *c* and $\mu_{0}$ are parameters (we used a=2, b=4.5, c=3.0, and $\mu_{0}=0.002$). Donating or receiving help changes the resource balance $\zeta_{i}$ (e.g., receiving the amount *u* increases $\zeta_{i}$), and thus the mortality rate, and this is illustrated in Fig. 2*A*.

## Supplementary Material

Appendix 01 (PDF)

## Data Availability

C++ source code for the individual-based simulations is available at GitHub, together with instructions for compilation on a Linux operating system: https://zenodo.org/doi/10.5281/zenodo.10396938 (40).
