# Supplementary material for: Social bond dynamics and the evolution of helping
Source: Proc Natl Acad Sci U S A. 2024 Mar 7;121(11):e2317736121. doi: 10.1073/pnas.2317736121 (PMC10945786; doi:10.1073/pnas.2317736121)
Supplement: Supplementary file 1 — Appendix 01 (PDF) [file pnas.2317736121.sapp.pdf]

Supporting Information for

Social bond dynamics and the evolution of helping

Olof Leimar and Redouan Bshary

Correspondence to Olof Leimar.
E-mail: olof.leimar@zoologi.su.se

This PDF file includes:

Supporting text
Tables S1 to S5
Figs. S1 to S10

Supporting Information Text

Overview. In order to achieve a self-contained presentation, we explain all model aspects here, including a model description and details of individual-based simulations, while referring to the presentation in the main text.

The Model

Individuals spend their lives in groups of size N, with a substructure of K subgroups available, implemented as places to go to. On average there are then $G = N/K$ individuals per subgroup. Time is divided into steps, where a time step is one day. At the start of a day, individuals choose which subgroup to join, and the resource state z_i of each individual i is $z_i = 1$ with probability $p_{\mathrm{s}i}$ (the individual succeeded in foraging) and $z_i = 0$ with probability $1 - p_{\mathrm{s}i}$. An individual's $p_{\mathrm{s}i}$ depends on its quality (see equation [S1] below). Only individuals i with $z_i = 0$ ask for help on a given day. They only ask help from individuals j they perceive have a resource state of $z_j = 1$. Individuals have a probability p_{d} of correctly detecting another's resource state.

Bond strength notation. The model combines two aspects of a social bond. One is the the accumulated history of helping between individuals i and j, which is denoted x_{ij}. The other represents the recent history of subgroup association between i and j, which is denoted ρ_{ij}. The effective (association adjusted) bond strength y_{ij}, influencing who to ask help from and how much help to donate, is the product of these: $y_{ij} = \rho_{ij} x_{ij}$. The qualitative effect of this formulation is that if one of two bonded individuals changes subgroup, causing a decrease in ρ_{ij}, if they again meet they will have a lower effective bond strength.

Genetically determined traits. An individual i has five genetically determined traits: (1) a bond strength learning rate α_i used to update x_{ij}, (2) a parameter β_i giving the degree of choosiness between subgroups with different estimated bond strengths, (3) an increment $\Delta y_{\mathrm{new}\,i}$ to the effective bond strength used when asking help from and donating to a new individual, (4) a helping asymptote $h_{\mathrm{a}i}$ and, (5) a helping sensitivity $h_{\mathrm{s}i}$ to the current helping balance.

Individual quality. Individuals vary in phenotypic quality q_i, which influences their probability $p_{\mathrm{s}i}$ of success in foraging. For individual i,

$$p_{\mathrm{s}i} = p_{\mathrm{s}0} + (1 - p_{\mathrm{s}0}) q_i, \qquad \text{[S1]}$$

with, for instance, $p_{\mathrm{s}0} = 0.8$. The quality q_i has a Beta distribution; we used a $\mathrm{B}(5, 5)$ distribution (so that $0 \le q_i \le 1$; see Figure S1A). The quality also influences the maximum amount of help an individual can provide to a partner in need. The maximum help is

$$h_{\max i} = h_{\mathrm{a}i}\Big(g_0 + (1 - g_0) q_i\Big), \qquad \text{[S2]}$$

with, for instance, $g_0 = 0.75$. A top quality individual ($q_i = 1$) can then donate a maximum amount of $h_{\max i} = h_{\mathrm{a}i}$.

Bond strength dynamics. For an individual i who requests help a given day, i.e. who has $z_i = 0$, there is updating of the bond strength. We assume that the starting value of bond strength is x_s, e.g., $x_\mathrm{s} = 1.25$, which should be large enough that at least some help is donated early in a partnership (see Fig. 1C). After a request from i to a particular j, i updates the value x_{ij} using the rate α_i and an asymptotic (maximum) bond strength x_a, e.g., $x_\mathrm{a} = 20.0$. So, with u_{ji} the amount of help from j to i, the update is

$$x'_{ij} = x_{ij} + \alpha_i u_{ji} \frac{x_\mathrm{a} - x_{ij}}{x_\mathrm{a} - x_\mathrm{s}}, \tag{S3}$$

where x'_{ij} is the updated value of x_{ij}. The donating individual j also updates its bond strength x_{ji}, as follows:

$$x'_{ji} = x_{ji} + \alpha_j u_{ji} \frac{x_\mathrm{a} - x_{ji}}{x_\mathrm{a} - x_\mathrm{s}}. \tag{S4}$$

These are the same as equations [1, 2] in the main text. See also Figs. 1A, 3A, S5A.

This bond strength dynamics is inspired by the Rescorla-Wagner (RW) model of classical conditioning. The interpretation is as follows. The asymptotic bond strength x_a corresponds to λ in the RW model, and might be considered a property of the partner, but for simplicity we let it be the same for all partners (we could have higher $x_{\mathrm{a}j}$ for partners j of higher quality). The rate α_i corresponds to the RW α. The interpretation of the amount of help u in equations [S3, S4] is analogous to the RW parameter β (or, instead, $u_{ji}/(x_\mathrm{a} - x_\mathrm{s})$ is analogous to RW β). In any case, having $x_\mathrm{a} - x_\mathrm{s}$ in the denominator simplifies the interpretation of the dynamics; $(x_\mathrm{a} - x_{ij})/(x_\mathrm{a} - x_\mathrm{s})$ starts out at 1 and then approaches 0 as the bond strength builds up.

Estimates of association. Each individual i maintains an estimate ρ_{ij} of the association between itself and and another group member j. The estimates are updated daily, when individuals are in their chosen subgroups. The update for i is

$$\rho'_{ij} = \rho_{ij} + \alpha_\mathrm{p}(\chi_{ij} - \rho_{ij}), \tag{S5}$$

where χ_{ij} is either one or zero and indicates whether i and j are in the same subgroup, and α_p is a subgroup association update parameter (e.g. $\alpha_\mathrm{p} = 0.1$). The starting value for ρ_{ij} is assumed to be zero.

Effective bond strength. The effective strength of the bond between i and j, as estimated by i, is the product of the estimated association ρ_{ij} between i and j and i's estimate x_{ij} of the accumulated exchange of help between i and j. So i's estimate of the strength of the bond between i and j is

$$y_{ij} = \rho_{ij} x_{ij}. \tag{S6}$$

Updating of the association between i and j is given in equation [S5], and the updating of accumulated helping between i and j appears in equations [S3, S4].

Requesting help. If individual i failed in foraging, so that $z_i = 0$, i will request help, choosing among available individuals j, i.e. those individuals that are in the same subgroup that day. Further, an individual i who is in need only considers others that it estimates to have succeeded in foraging, i.e., estimated to have $z_j = 1$, where the estimate is correct with probability p_d (e.g., $p_\mathrm{d} = 0.98$). If there is more than one such alternative, individual i uses a soft-max procedure on the effective bond strengths $y_{ij} = \rho_{ij} x_{ij}$ to choose the one to ask help from. In addition, individual i takes into account if it has previously received help from j, or if j is a new donor individual. For an established partner, the probability of requesting help from j is proportional to

$$\exp(y_{ij}). \quad [\mathrm{S7}]$$

See Figure 1B and equation [3] for an illustration with choice between two individuals. For a new donor j, the amount $\Delta y_{\mathrm{new}\,i}$ is added to the effective bond strength, leading to the probability of requesting help from j being proportional to

$$\exp(y_{ij} + \Delta y_{\mathrm{new}\,i}). \quad [\mathrm{S8}]$$

An individual in need can ask help from more than one of the available individuals. In the model, individuals have the opportunity to ask for help twice, from different individuals among the available ones in the current subgroup.

Group substructure and choice of subgroups. The model allows for the possibility that a group is structured into temporary subgroups, which we identify with places, e.g., resting places. There are K subgroups and each day each group member chooses which subgroup to join.

We assume that the probability for an individual to select a particular subgroup is influenced by an estimated effective bond strength experienced by the individual in that subgroup, but that there is a small probability ϵ_p that the individual ends up in a random subgroup (e.g., $\epsilon_\mathrm{p} = 0.05$). Further, to limit the outcome that all individuals in the group select the same subgroup, we assume that there is density dependence in the probability of selecting a subgroup. Let $\hat{n}_{ik}$ be an estimate by individual i of the density (number of individuals) in subgroup k, and $\hat{y}_{ik}$ an estimate by i of the effective bond strength of subgroup k. The update rate α_p is used to update the estimated densities,

$$\hat{n}'_{ik} = \hat{n}_{ik} + \alpha_\mathrm{p}(n_k - \hat{n}_{ik}), \quad [\mathrm{S9}]$$

where k is the current subgroup for individual i and n_k is the actual current density in subgroup k. Similarly, the subgroup bond strength is updated as

$$\hat{y}'_{ik} = \hat{y}_{ik} + \alpha_\mathrm{p}(y_{ij} - \hat{y}_{ik}), \quad [\mathrm{S10}]$$

where $y_{ij} = \rho_{ij} x_{ij}$ is the effective bond strength to another individual j currently in subgroup k. The update in this equation is performed for each other individual j in the current subgroup.

The probability that individual i selects subgroup k is then, with probability $1 - \epsilon_\mathrm{p}$, proportional to

$$\frac{\exp\Big(\beta_i \hat{y}_{ik}\Big)}{1 + \exp\Big(\beta_\mathrm{d}(\hat{n}_{ik} - G)\Big)}, \quad [\mathrm{S11}]$$

where β_i is the subgroup choosiness trait of i, and β_d is a parameter (e.g., $\beta_\mathrm{d} = 4.0$). The parameter $G = N/K$ acts as a carrying capacity for subgroup k. With probability ϵ_p, however, the choice of subgroup is random. For $K = 2$ and equal density estimates for both subgroups, the result is equation [4] in the main text (see Figure 1B for an illustration).

Helping amounts. The effective bond strength y_{ij}, as estimated by i, is taken into account by i when deciding on the amount of help to provide when j requests it. In the model, this is expressed as a helping function

$$H_y(y_{ij}) = \frac{1}{1 + \exp\Big(-d(y_{ij} - y_0)\Big)}, \quad \text{[S12]}$$

where d and y_0 are parameters (e.g., $d = 8.0$ and $y_0 = 1.5$). The above is the same as equation [5] in the main text. This holds for an established partner j that i has donated to before, but for a new recipient j, the (positive or negative) amount $\Delta y_{\mathrm{new}\,i}$ is added to the effective bond strength y_{ij}, resulting in the value $H_y(y_{ij} + \Delta y_{\mathrm{new}\,i})$, and this is illustrated in Fig. 1C.

The amount of help provided also depends on the total helping balance w_{ij}. The total helping balance between i and j is the sum of the amounts u_{ji} of help previously received from j minus the sum of the amounts u_{ij} previously donated by i to j. Further, let ζ_i denote the current day resource balance for individual i. The within-day dynamics of this variable is

$$\zeta_i' = \zeta_i + u_{ji}, \quad \text{[S13]}$$

after i has received the amount u_{ji} from j, and

$$\zeta_i' = \zeta_i - u_{ij}, \quad \text{[S14]}$$

after i has donated the amount u_{ij} to j. We assume $\zeta_i = 0.5q_i$ at the start of the day. The reason this variable is needed is that an individual i might be involved in several exchanges of help on the same day. In the model, the influence of q_i, w_{ij}, z_i, and ζ_i on the helping amount is expressed as the function

$$H_w(q_i, w_{ij}, z_i, \zeta_i) = \frac{h_{\mathrm{a}i}\Big(g_0 + (1 - g_0)q_i\Big)}{1 + \exp(-h_{\mathrm{s}i}w_{ij} - bz_i - c\zeta_i)}, \quad \text{[S15]}$$

where the parameters b and c are the same as in the mortality function below (in equation [S17], e.g., $b = 4.5$ and $c = 3.0$). This is the same as equation [6] in the main text. Also note that the numerator in this equation is the value $h_{\max i}$ from equation [S2]. See Figure 1D for an illustration of the function. The amount donated from i to j is the product of the two functions:

$$u_{ij} = H_y(y_{ij})H_w(q_i, w_{ij}, z_i, \zeta_i), \quad \text{[S16]}$$

or, for a new recipient, the same but adding the amount $\Delta y_{\mathrm{new}\,i}$ to y_{ij}.

The above means that the social bond mechanism implements a form of state-dependent reciprocity. The reciprocity is expressed in how the amount of help is influenced by the effective bond strength y_{ij} and by the helping balance w_{ij}. Depending on how $\Delta y_{\mathrm{new}\,i}$ evolves, new individuals can be treated differently from established partners. There is also an influence of the quality q_i on the amount of help an individual provides, as seen from equations [S2] and [S15].

Survival effects. Survival is the only fitness component acting in the model. The (daily) rate of mortality depends on the foraging success state z_i and the current-day resource balance ζ_i. The rate of mortality for an individuals i is then

$$\mu(z_i, \zeta_i) = \frac{1 - \mu_0}{1 + \exp\Big(a + bz_i + c\zeta_i\Big)} + \mu_0, \qquad [S17]$$

where a, b, c, and μ_0 are parameters. This is the same as equation [7] in the main text. We used the parameter values $a = 2.0$, $b = 4.5$, and $c = 3.0$, which means that succeeding in foraging ($z_i = 1$ vs. $z_i = 0$) corresponds to receiving an amount of $4.5/3 = 1.5$ units of help. The parameter μ_0 is a background rate of mortality, irrespective of the individual's resource state (we used $\mu_0 = 0.002$). The resulting effects on mortality of donating and receiving help are illustrated in Fig. 2A.

In order to evaluate marginal costs and benefits of donating and receiving help, we need the partial derivative of μ with respect to ζ:

$$\frac{\partial \mu(z_i, \zeta_i)}{\partial \zeta_i} = -(1 - \mu_0) \frac{c \exp\Big(a + bz_i + c\zeta_i\Big)}{\Big(1 + \exp\Big(a + bz_i + c\zeta_i\Big)\Big)^2}. \qquad [S18]$$

The marginal cost for an individual i with $z_i = 1$ and quality q_i, at the point of donating the amount u_{ij}, is minus this partial derivative evaluated at $z_i = 1$ and $\zeta_i = 0.5q_i - u_{ij}$. The corresponding marginal benefit for an individual j with $z_j = 0$ and quality q_j, at the point of receiving the amount u_{ij}, is minus this partial derivative evaluated at $z_j = 0$ and $\zeta_j = 0.5q_i + u_{ij}$. Using this to examine the marginal benefit-cost ratios for average quality individuals ($q_i = 0.5$), we find that the ratio for a very small amount is quite large, around 80, whereas at the point of transferring an amount $u = 0.4$, the ratio is 7.9. The ratio then decreases sharply as the amount gets larger, which can be deduced from Fig. 2A.

Individuals that die are replaced through reproduction by surviving individuals. This happens at intervals of T days (e.g., $T = 20$). To avoid effects of local relatedness, parents to a new individual are randomly drawn from the global population.

Model variants. *Visitors between groups.* For this model variant, a number of randomly selected pairs of groups exchange visitors, with results in Fig. S6 and Table S2 (in this case, 20 random pairs out of 250 groups exchanged visitors for a given 20 day interval). For a visiting exchange, each individual in a group had a 50% chance of becoming a visitor in the paired group. We assume that individuals distinguish between visitors and residents, such that individuals have an additional trait $\Delta y_{\text{vis},i}$, with a similar function as $\Delta y_{\text{new},i}$, but applied to visitors (see Table S2). A further possibility, which we did not implement, might be to let some of the visitors stay in the new group, thus becoming residents.

No individual recognition. This model variant can be regarded as investigating the evolution of generalized reciprocity, with continuous variation in helping amounts, with results in Fig. S7 and Table S3. We assume that an individual treats all other individuals as the same, with full association ($\rho_{ij} = 1$), an already established bond (we used $x_{ij} = 2.5$), and a helping balance w_{ij} formed from all received and donated help. In this way, the helping function H_w in equations [6, S15] and Fig. 1D regulates the exchange of help.

Times asking help and treatment of new partner. To investigate the consequences of individuals in need having the opportunity to ask for three times (instead of twice), we ran a simulation otherwise corresponding to case 1 in Table S1 ($N = 24$ and $G = 4$). The results appears as case 17 in Table S4. Comparing with case 1, the main evolutionary effect of this change is that the bond learning rate α_i evolves to a higher value. Finally, we made the change from case 1 that a partner is treated as new for up to two times donating or asking help (instead of once). The results appears as case 18 in Table S4 and, comparing with case 1, the main evolutionary effect is that α_i evolves to a lower value.

Details of individual-based simulations. There are two issues that are helpful to keep in mind when performing evolutionary simulations for the model. First, the cost of donating help might be fairly low, which means that selection can be relatively weak. Second, the model has five traits, which also means that the strength of selection on each particular trait can be weak. It is then important for there to be ample and independent amounts of genetic variation in each trait, so that evolutionary change can proceed efficiently. To achieve this in a simple way, we assume that each trait is determined by an unlinked haploid locus, but with sexual reproduction and free recombination, as follows. To produce a haploid offspring, two parents are chosen to form a diploid from copies of their haploid genomes, and the haploid offspring is then formed with recombination and mutation from this diploid (thus, adult individuals can be regarded as gametophytes). Alleles mutate with a fairly high probability of 0.002, and the mutational increments have Laplacian (bi-exponential) distributions (these distributions have long tails), with standard deviations for a locus ranging from 0.10 down to 0.04, chosen so that simulations could readily locate evolutionary equilibria. We use lower and upper limits for allelic (trait) values, with a fairly high upper limit of 8.0 to allow traits to evolve to large values (see Table S1).

Reproduction occurs at intervals of T days ($T = 20$), at which time individuals that have died during the interval are replaced by new offspring, with parents randomly selected from the population. Population size for a simulation is at least 4000 (e.g., 175 groups, each with $N = 24$ individuals, results in a population size of 4200). For each case reported in Tables S1 to S4, after having reached evolutionary equilibrium, successive simulations were performed over $200,000$ days (approximately 700 generations), repeated at least 100 times, to estimate means and standard deviations of the traits at evolutionary equilibrium.

Table S1. Trait values (mean ± SD over 100 simulations, each over $100\,000$ days) for 7 different cases of individual-based evolutionary simulations of helping with social bonds. The parameters that vary between cases are the group size (N) and the average number of individuals per subgroup (G). For case 7, one trait was kept fixed.

case	α_i	β_i	$\Delta y_{\text{new } i}$	$h_{\text{a}i}$	$h_{\text{s}i}$
1: $N = 24, G = 4$	0.97 ± 0.13	7.89 ± 0.09	7.80 ± 0.12	0.47 ± 0.02	4.50 ± 0.45
2: $N = 24, G = 6$	0.60 ± 0.06	7.86 ± 0.10	7.69 ± 0.19	0.46 ± 0.02	5.31 ± 0.32
3: $N = 24, G = 8$	0.60 ± 0.05	7.83 ± 0.11	7.66 ± 0.28	0.41 ± 0.02	4.85 ± 0.45
4: $N = 8,\ G = 8$	0.32 ± 0.04	–	3.59 ± 0.70	0.52 ± 0.02	5.82 ± 0.64
5: $N = 16, G = 16$	0.69 ± 0.10	–	5.68 ± 0.76	0.31 ± 0.02	5.75 ± 0.60
6: $N = 24, G = 24$	1.41 ± 0.19	–	6.99 ± 0.55	0.01 ± 0.01	6.10 ± 0.76
7: $N = 24, G = 24$	7.90 ± 0.07	–	**-1**	0.65 ± 0.02	4.05 ± 0.26

Table S2. Trait values for a simulation with the model variant where groups sometimes exchange temporary visitors, with $N = 16$ and $G = 8$.

case	α_i	β_i	$\Delta y_{\text{new } i}$	$\Delta y_{\text{vis } i}$	$h_{\text{a}i}$	$h_{\text{s}i}$
8:	0.61 ± 0.06	7.29 ± 0.54	7.52 ± 0.31	-3.66 ± 0.27	0.46 ± 0.01	7.01 ± 0.43

Table S3. Trait values for 8 cases of individual-based evolutionary simulations of helping without individual recognition. The group size for cases 9 and 10 is $N = 2$ ($h_{\text{s}i}$ is fixed at 0 for case 10), and the other correspond to cases 1 to 6 in Table S1.

case	α_i	β_i	$\Delta y_{\text{new } i}$	$h_{\text{a}i}$	$h_{\text{s}i}$
9: $N = 2,\ G = 2$	–	–	–	0.80 ± 0.04	2.85 ± 0.47
10: $N = 2,\ G = 2$	–	–	–	0.02 ± 0.02	**0**
11: $N = 24, G = 4$	–	–	–	0.02 ± 0.01	4.42 ± 0.76
12: $N = 24, G = 6$	–	–	–	0.02 ± 0.01	3.79 ± 0.65
13: $N = 24, G = 8$	–	–	–	0.02 ± 0.01	4.67 ± 0.57
14: $N = 8,\ G = 8$	–	–	–	0.27 ± 0.05	3.52 ± 0.33
15: $N = 16, G = 16$	–	–	–	0.01 ± 0.01	1.90 ± 0.35
16: $N = 24, G = 24$	–	–	–	0.02 ± 0.02	4.56 ± 0.79

Table S4. Trait values for two model variants with $N = 24$ and $G = 4$. Compared to case 1 in Table S1, for case 17 individuals in need can ask help tree times, and for case 18 a partner is treated as new for two donations or times asking help.

case	α_i	β_i	$\Delta y_{\text{new } i}$	$h_{\text{a}i}$	$h_{\text{s}i}$
17 (ask help 3 times):	2.53 ± 0.24	7.88 ± 0.11	7.73 ± 0.24	0.44 ± 0.01	5.54 ± 0.28
18 (new partner 2 rounds):	0.50 ± 0.06	7.59 ± 0.20	7.56 ± 0.33	0.45 ± 0.02	5.28 ± 0.26

Table S5. Definitions and notation for the model.

notation	definition or explanation
N	size of local group
K	number of subgroups (places) available to group members
G	expected number individuals per subgroup; $G = N/K$
z_i	current foraging success state (0/1) for individual i
q_i	phenotypic quality $(0 < q_i < 1)$ for i; see Fig. S1A
ζ_i	current resource balance for i; $\zeta_i = 0.5 q_i$ at start of day
$p_{\mathrm{s}i}$	probability of foraging success; $p_{\mathrm{s}i} = p_{\mathrm{s}0} + (1 - p_{\mathrm{s}0}) q_i$
$p_{\mathrm{s}0}$	parameter; $p_{\mathrm{s}0} = 0.8$ in simulations
p_{d}	probability of detecting another's state z_j; $p_{\mathrm{d}} = 0.98$
x_{ij}	bond strength between individuals i and j
x_{s}	starting value of x_{ij}: $x_{\mathrm{s}} = 1.25$
x_{a}	asymptotic (maximum) value of x_{ij}: $x_{\mathrm{a}} = 20.0$
ρ_{ij}	subgroup association between i and j
$y_{ij} = \rho_{ij} x_{ij}$	effective (association adjusted) bond strength
$\hat{y}_{ik}$	average effective bond strength for i to subgroup k
α_i	bond strength learning rate for individual i
β_i	sensitivity to among subgroup bond strength differences
$\Delta y_{\mathrm{new}\, i}$	bond strength increment for i for a new partner
$h_{\mathrm{a}i}$	asymptotic helping amount for i
$h_{\mathrm{s}i}$	helping balance sensitivity for i
$\Delta y_{\mathrm{vis}\, i}$	between-group version of $\Delta y_{\mathrm{new}\, i}$ for model with visitors
$\mu(z_i, \zeta_i)$	rate (per day) of mortality as a function of z_i and ζ_i
a, b, c, μ_0	parameters for μ; $a = 2.0,\ b = 4.5,\ c = 3.0,\ \mu_0 = 0.002$
$u_{ij} = H_y H_w$	amount of help donated by i to j
$H_y(y_{ij})$	help as function of y_{ij}
d, y_0	parameters for H_y; $d = 8.0,\ y_0 = 1.5$
w_{ij}	helping balance: received minus donated between i and j
$H_w(q_i, w_{ij}, z_i, \zeta_i)$	help as function of $q_i,\ w_{ij},\ z_i$, and ζ_i
g_0, b, c	parameters for H_w; $g_0 = 0.75,\ b = 4.5,\ c = 3.0$
ϵ_{p}	probability of ending up in a random subgroup; $\epsilon_{\mathrm{p}} = 0.05$
α_{p}	rate of updating of estimates $\rho_{ij},\ \hat{y}_{ik}$, and $\hat{n}_{ik}$; $\alpha_{\mathrm{p}} = 0.1$
β_{d}	sensitivity to density estimate in choice of subgroup; $\beta_{\mathrm{d}} = 4.0$
$\hat{n}_{ik}$	density estimate by i for subgroup k

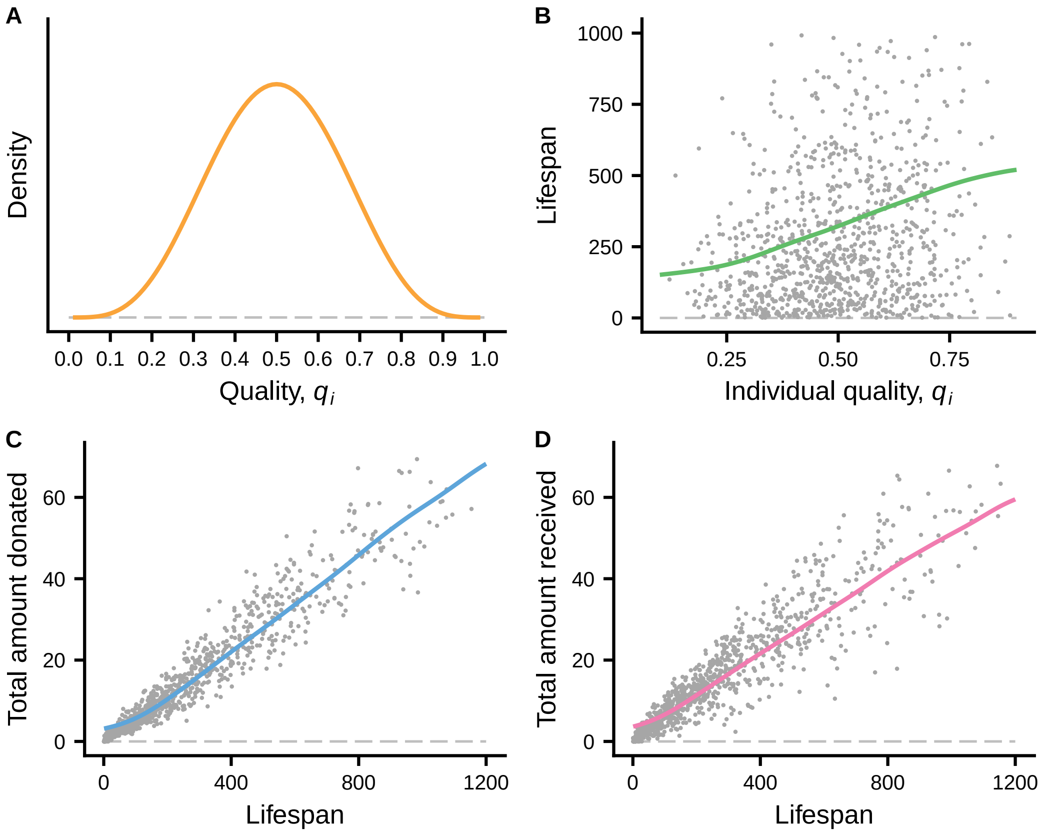

Fig. S1. (A) The distribution of individual quality (q_i) is given by a beta distribution with shape parameters equal to 5, B$(5, 5)$. **(B)** Lifespan vs. individual quality for a random sample of 1000 individuals from those analysed in Fig. 2, together with the fitted curve in Fig. 2B. **(C)** and **(D)** Total amounts of help donated and received vs. lifespan for the sample, together with the fitted curves from Fig. 2C.

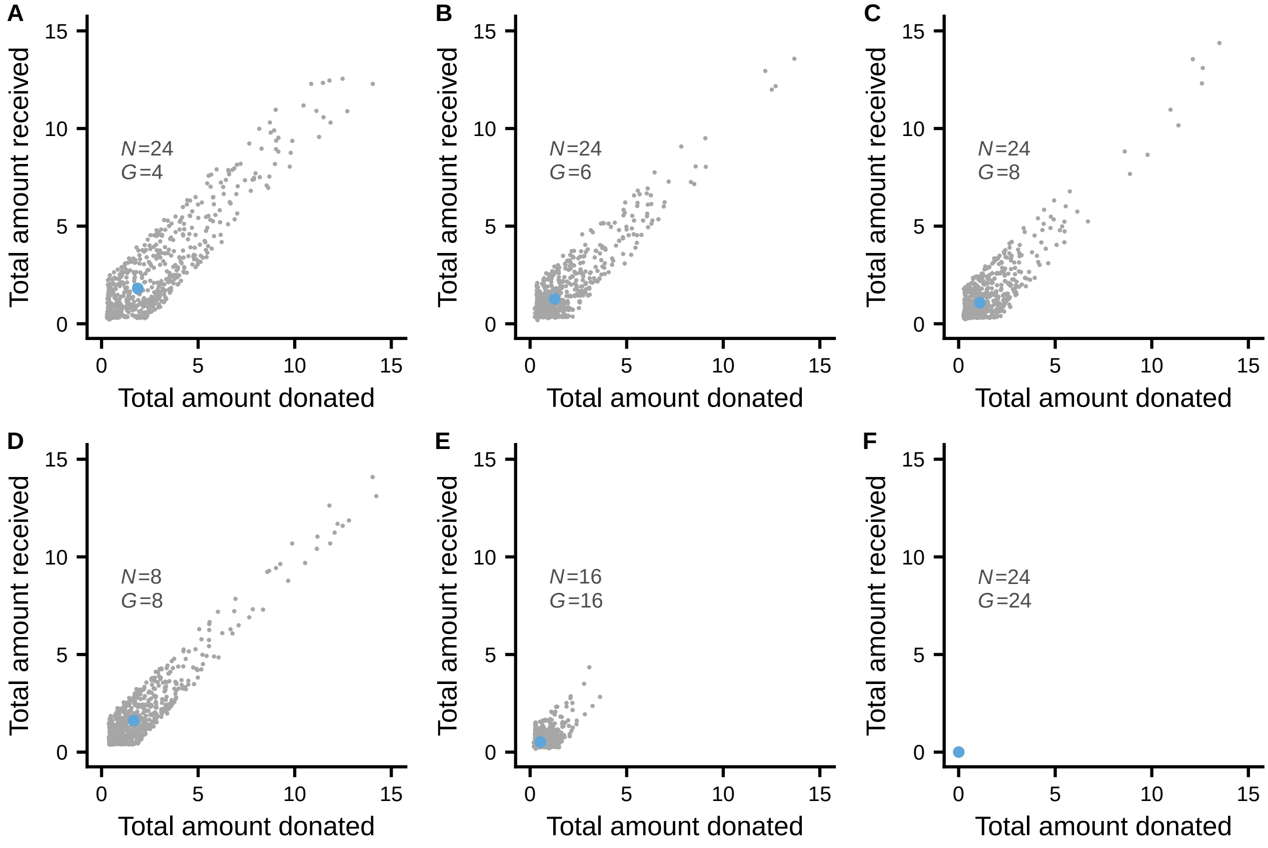

Fig. S2. Effect of the size of the social neighbourhood on the total amount of help in relationships with at least one exchange in each direction. The grey points in the panels show total received vs. total donated in random samples (of size 1000) of relationships from evolutionary simulations. The larger blue points show the sample means. Each simulation (used in Fig. 5) has a population size of at least 4000, split into groups of size N. Each group has one or more subgroups where individuals can associate, and G is the expected number of individuals in a subgroup. Panels **(A)**, **(B)**, and **(C)** show cases with group size $N = 24$, split up into 6, 4, or 3 subgroups (so that $G = 4, 6, 8$), and panels **(D)**, **(E)**, and **(F)** show cases with different group sizes $N = 8, 16, 24$ and a single subgroup (so that $G = N$). Note that helping evolved to near zero in panel **(F)**. Each simulation was run at evolutionary equilibrium for 4000 periods, and the random samples are of individuals born in the earlier part of this interval (thus including those with long lifespans).

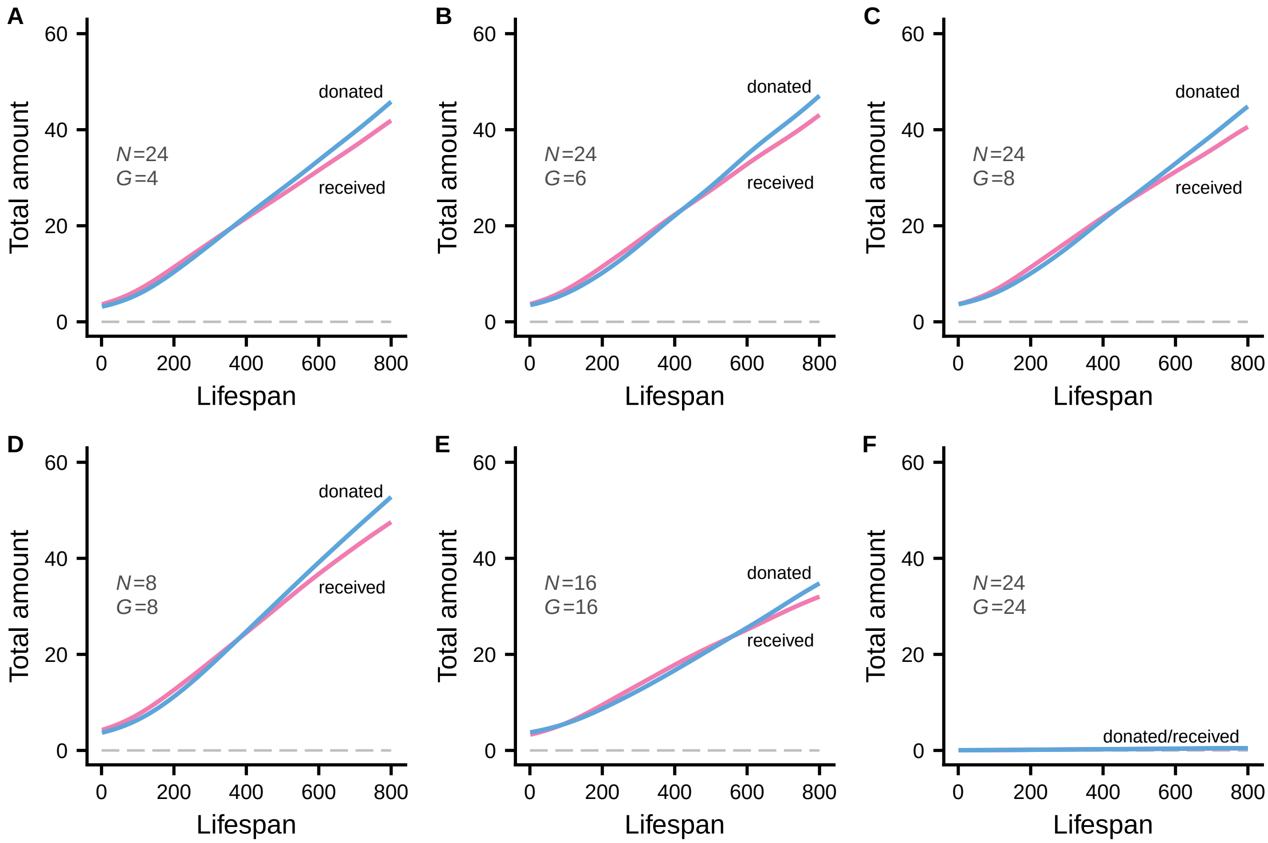

Fig. S3. Effect of the size of the social neighbourhood on the expected total amount of help donated and received (to and from all other individuals) as a function of an individual's lifespan. Each simulation (used in Fig. 5) has a population size of at least 4000, split into groups of size N. Each group has one or more subgroups where individuals can associate, and G is the expected number of individuals in a subgroup. Panels **(A)**, **(B)**, and **(C)** show cases with group size $N = 24$, split up into 6, 4, or 3 subgroups (so that $G = 4, 6, 8$), and panels **(D)**, **(E)**, and **(F)** show cases with different group sizes $N = 8, 16, 24$ and a single subgroup (so that $G = N$). Note that helping evolved to near zero in panel **(F)**. Each simulation was run at evolutionary equilibrium for 4000 periods, and the random samples are of individuals born in the earlier part of this interval (thus including those with long lifespans). The curves are kernel smoothing fits and are based on ca 12 000 individuals.

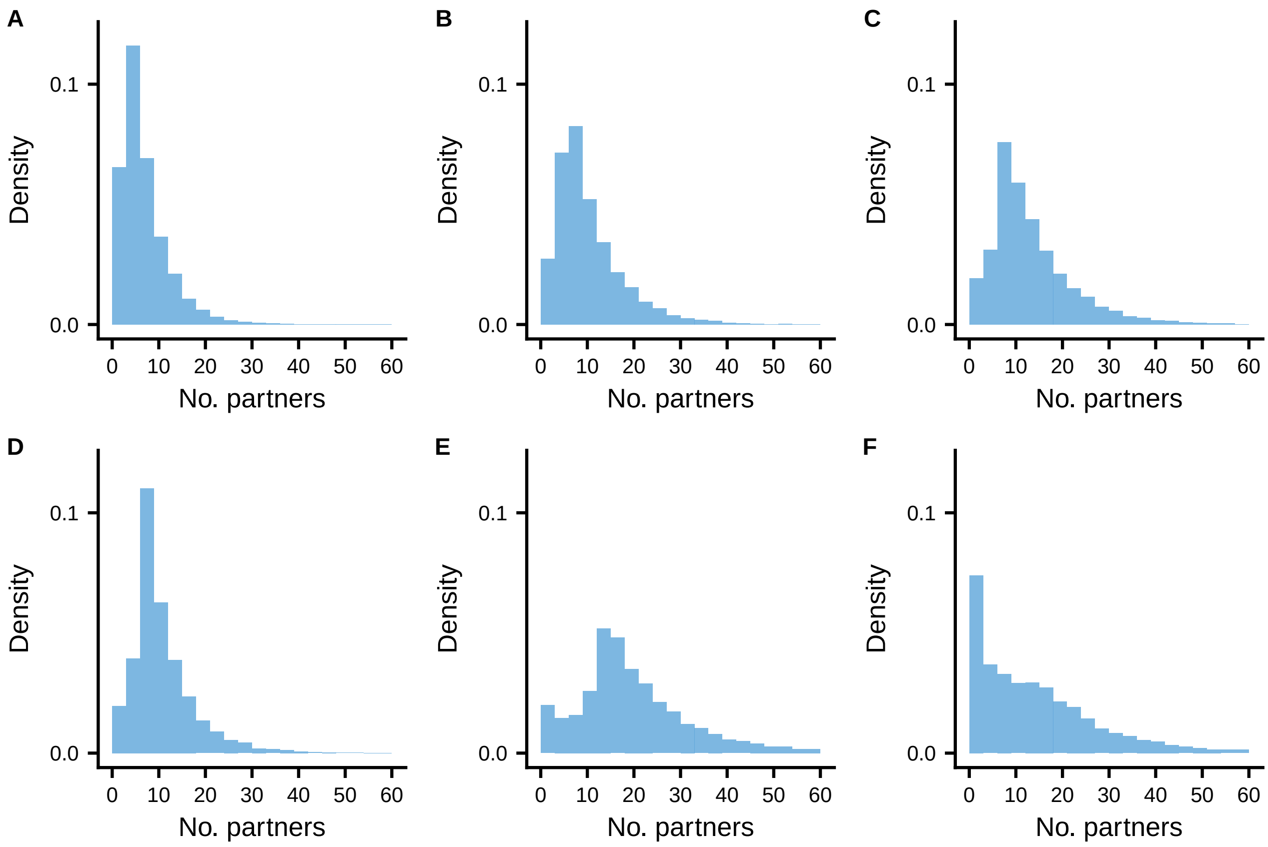

Fig. S4. Effect of the size of the social neighborhood on the distribution of lifetime number of established partners (i.e., those with at least one exchange in each direction). Each simulation (used in Fig. 5) has a population size of at least 4000, split into groups of size N. Each group has one or more subgroups where individuals can associate, and G is the expected number of individuals in a subgroup. Panels **(A)**, **(B)**, and **(C)** show cases with group size $N = 24$, split up into 6, 4, or 3 subgroups (so that $G = 4, 6, 8$), and panels **(D)**, **(E)**, and **(F)** show cases with different group sizes $N = 8, 16, 24$ and a single subgroup (so that $G = N$). Note that, comparing with Figs. 5 and S2, there is a quality-quantity trade off in established partnerships: situations with more helping are characterized by fewer but more valuable established partnerships (with more total help exchanged). Note also that helping amounts evolved to near zero in panel **(F)**.

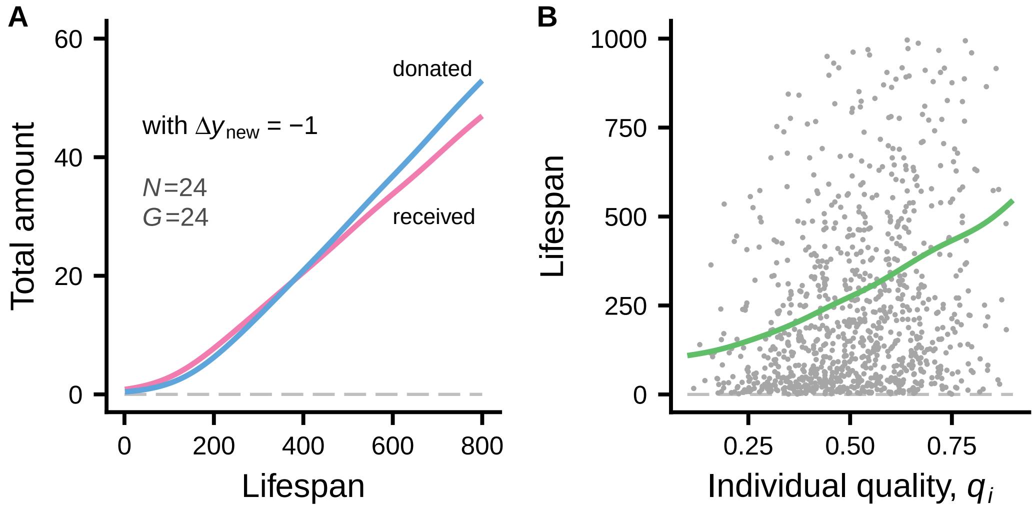

Fig. S5. Evolution of helping when there is a tendency to avoid forming bonds with new individuals. This is achieved by simulating evolution with the trait $\Delta y_{\mathrm{new}\ i}$ fixed at -1. **(A)** Total amounts of help donated and received as a function of lifespan. **(B)** Lifespan vs. individual quality for a random sample of 1000 individuals, together with a fitted curve. The simulation has a population size of 4200, split into groups of size $N = 24$ with a single subgroup ($G = 24$). The curves are kernel smoothing fits and are based on ca 12 000 individuals, including those with long lifespans.

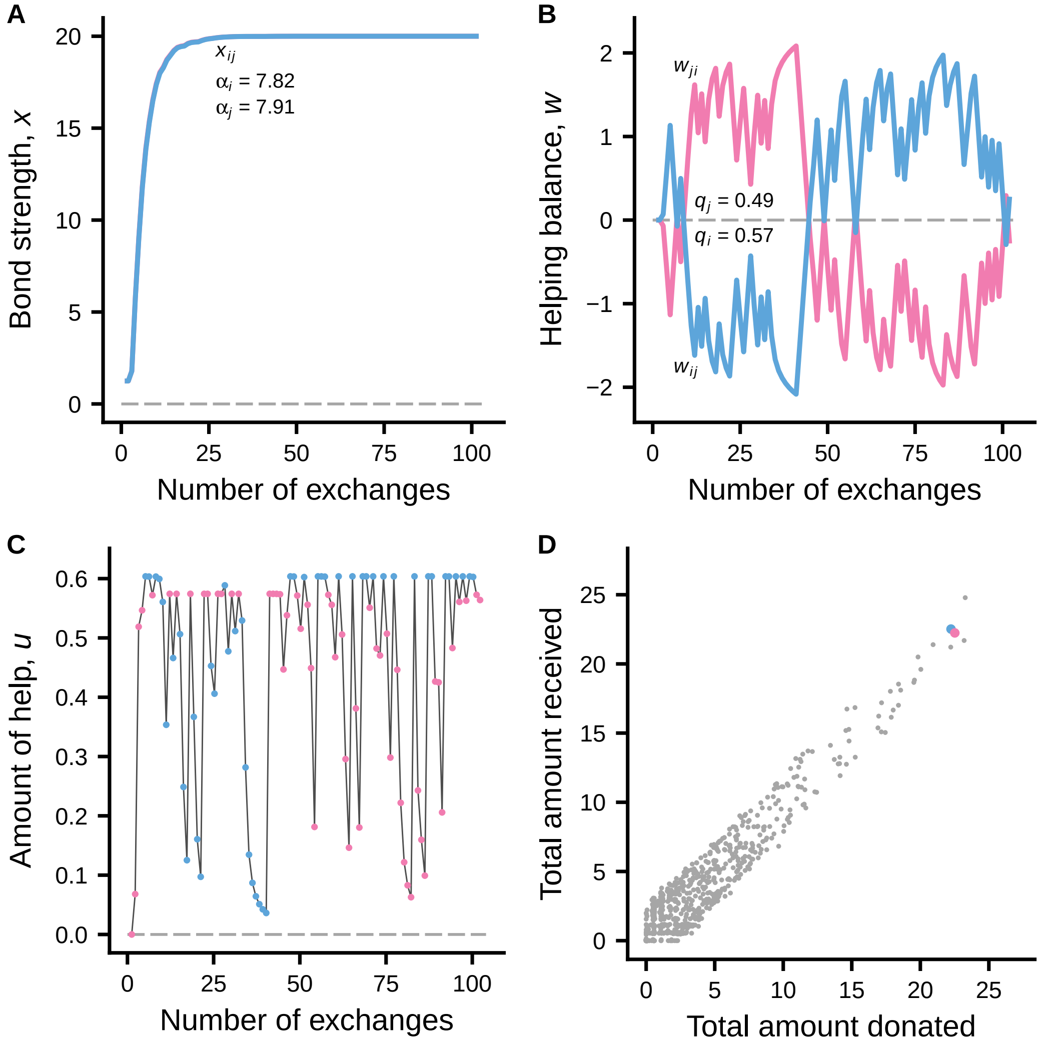

Fig. S6. Example of bond dynamics and helping for a pair i and j with many helping exchanges, from the simulation with the trait $\Delta y_{\text{new}\,i}$ fixed at -1 (see Fig. S5). In the panels, blue and red indicate i and j. **(A)** The build-up of bond strength x_{ij} for i (which overlaps with x_{ji} for j). The genetically determined bond strength learning rates α_i and α_j are shown in the panel. **(B)** The total helping balance w_{ij} for i and w_{ji} for j, together with the phenotypic quality values q_i and q_j for the example. **(C)** The amounts of help u_{ij} and u_{ji} donated by one individual to the other. **(D)** The colour coded points show the total amounts donated and received between i and j. The additional grey points show a sample of 1000 partnerships from those with helping at least once in each direction. The data come from the simulation shown as case 7 in Table S1.

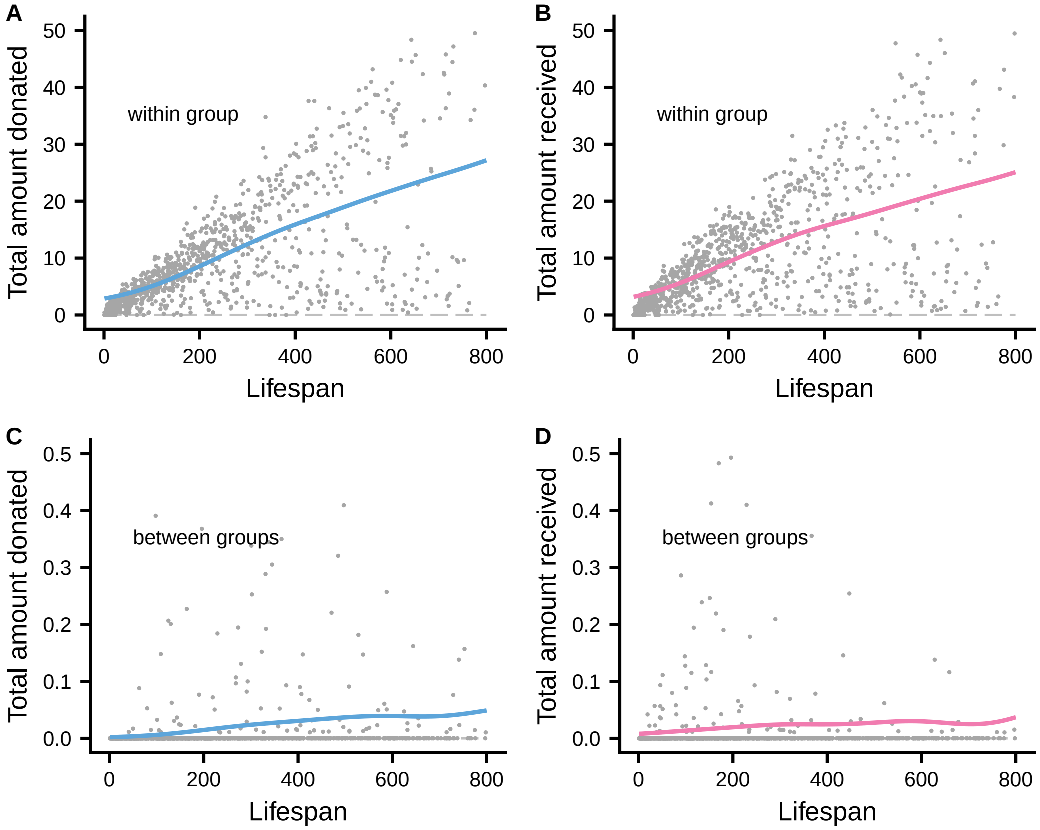

Fig. S7. Total amounts donated and received as a function of lifespan for the model variant where some groups exchange visitors over a short period (20 days). The visitors subsequently return to their original group. **(A)** and **(B)** Total amounts of help donated and received between members of the same original group as a function of lifespan. **(C)** and **(D)** Total amounts of help donated and received between members of different original groups as a function of lifespan. Note the difference in scale on the y-axes.

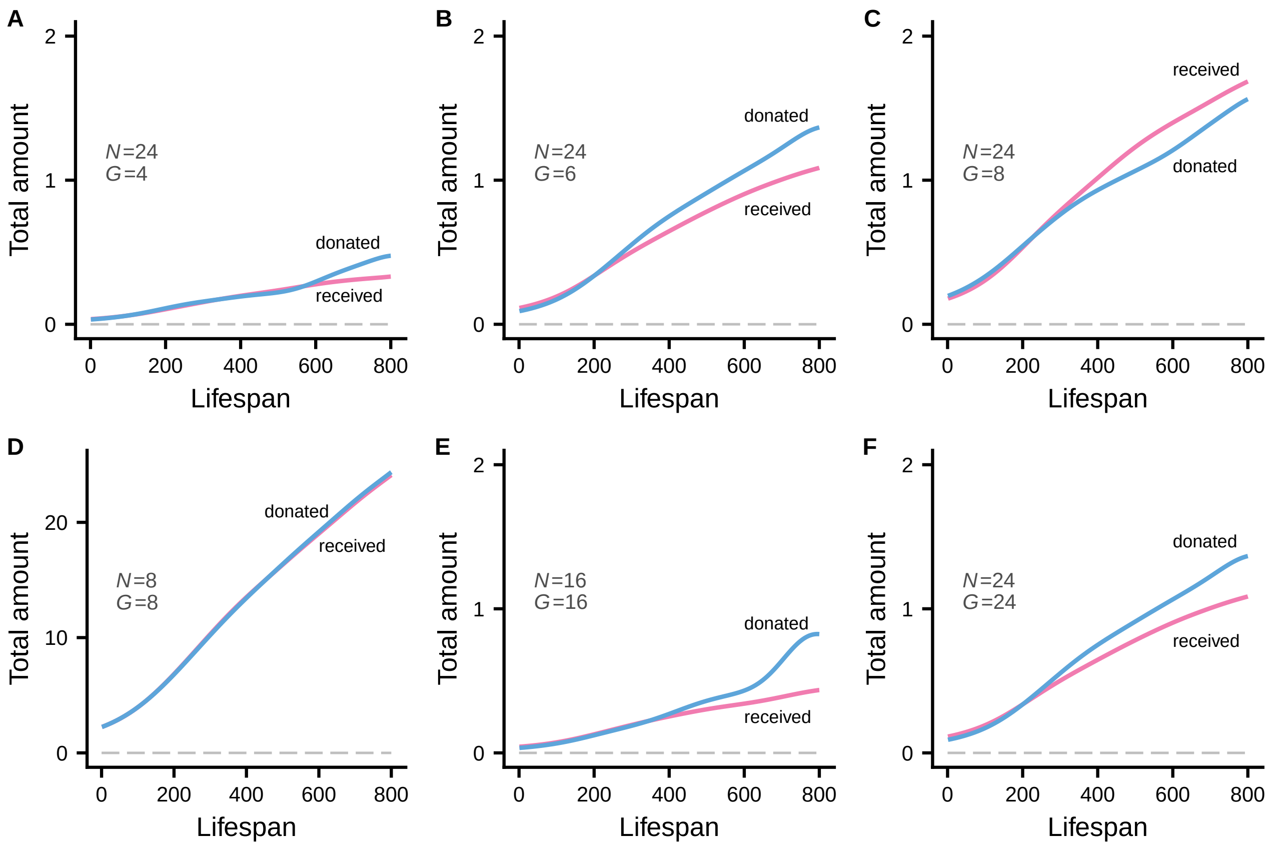

Fig. S8. Effect of the size of the social neighbourhood, as in panels **(A)**, **(B)**, **(C)** of Fig. S3, but for evolutionary simulations without individual recognition. Note that the scale on the y-axis differs from that in Fig. S3. With individual recognition (Fig. S3), the amounts of help exchanged are much larger than here. Each simulation has a population size of 4200, split into groups of size $N = 24$, with one or more subgroups, and G is the expected number of individuals in a subgroup. Removing individual recognition means that an individual treats all other individuals as the same, having a social bond to this collective, and there is no basis for preferring one subgroup to another. The curves are kernel smoothing fits and are based on ca 12 000 individuals. Note the different scale on the y-axes in panel **(D)**.

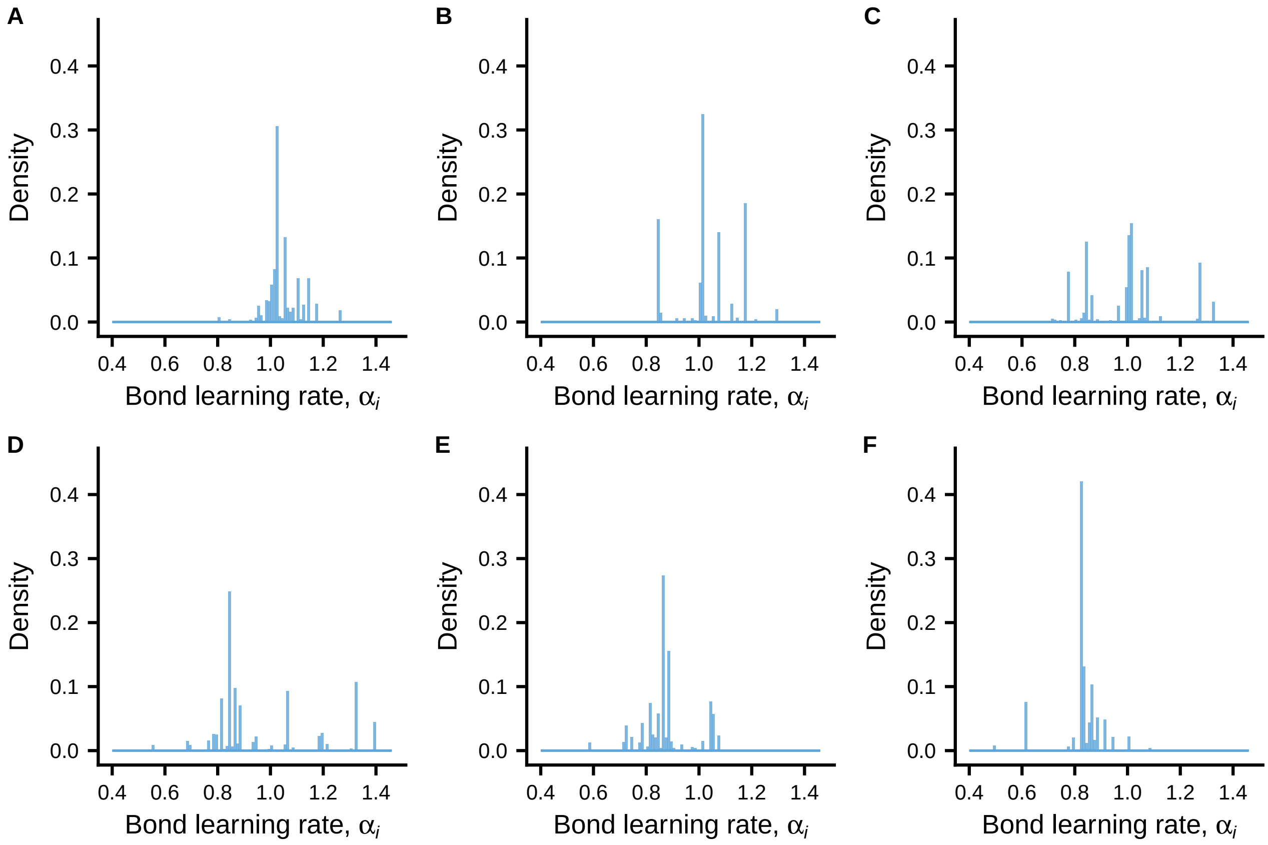

Fig. S9. Examples of trait distributions during individual-based simulations for case 1 in Table S1. The different panels **(A)** to **(F)** show the distributions of the bond strength learning rate α_i, at approximately 700 generations apart.

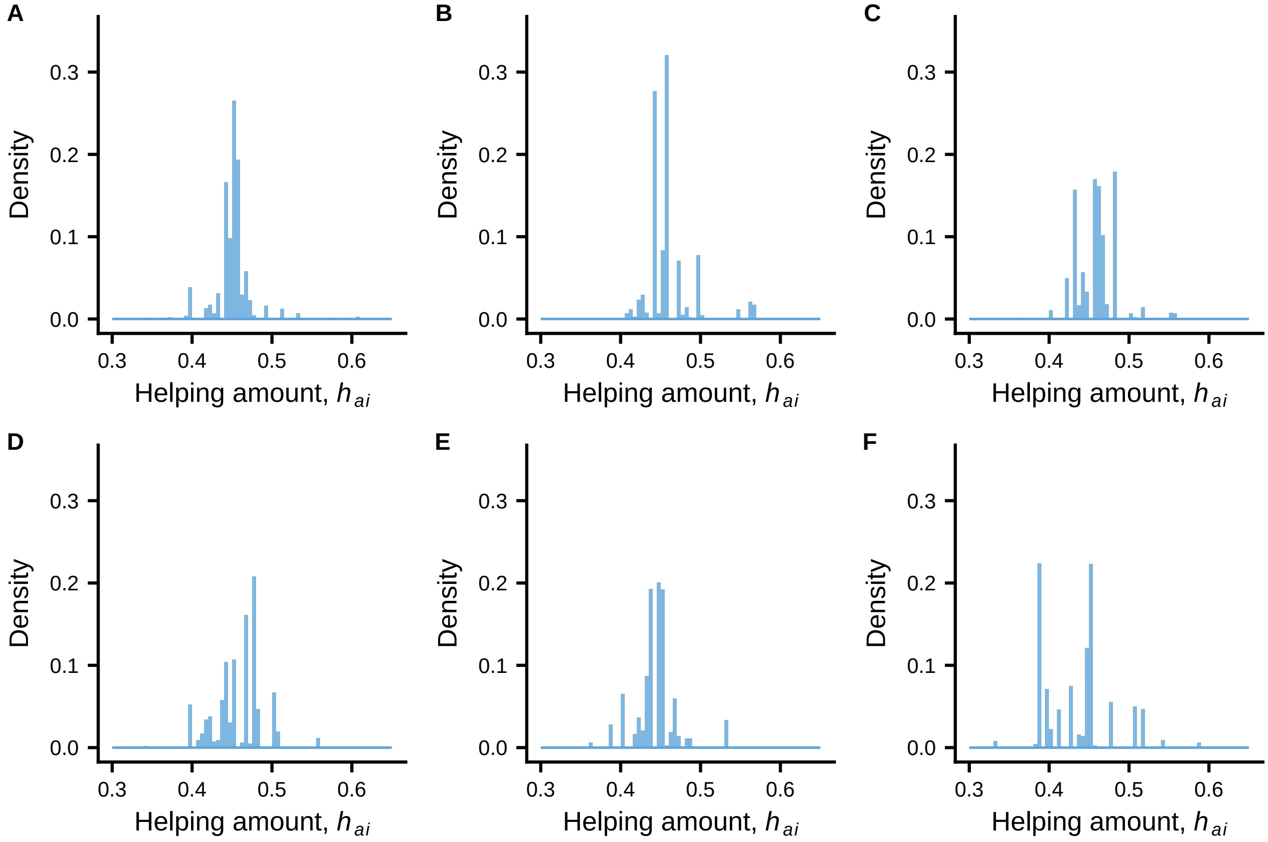

Fig. S10. Examples of trait distributions during individual-based simulations for case 1 in Table S1. The different panels **(A)** to **(F)** show the distributions of the maximum helping amount h_{ai}, at the same time points as in Fig. S9, approximately 700 generations apart.
